# Associating EEG functional networks and the effect of sleep deprivation as measured using psychomotor vigilance tests

**DOI:** 10.1038/s41598-024-78814-4

**Published:** 2024-11-14

**Authors:** Sophie L. Mason, Leandro Junges, Wessel Woldman, Suzanne Ftouni, Clare Anderson, John R. Terry, Andrew P. Bagshaw

**Affiliations:** 1 Centre for Systems Modelling and Quantitative Biomedicine, University of Birmingham, Birmingham, B15 2TT UK; 2 Centre for Human Brain Health, College of Life and Environmental Sciences, University of Birmingham, Birmingham, B15 2TT UK; 3 Neuronostics Limited, Engine Shed, Station Approach, Bristol, UK; 4 Turner Institute for Brain and Mental Health, School of Psychological Sciences, Monash University, Clayton, VIC 3800 Australia

**Keywords:** Graph theory, Functional connectivity, Functional networks, Sleep deprivation, EEG, Psychomotor vigilance test (PVT), Circadian mechanisms, Sleep deprivation, Network models

## Abstract

People are routinely forced to undertake cognitive challenges under the effect of sleep deprivation, due to professional and social obligations forcing them to ignore their circadian clock. However, low intra-individual and high inter-individual differences in behavioural outcomes are known to occur when people are sleep deprived, leading to the conclusion that trait-like differences to sleep deprivation could explain the differing levels of resilience. Within this study we consider if trait-like resilience to sleep deprivation, measured using psychomotor vigilance tests over a 40 h protocol, could be associated with graph metrics (mean node strength, clustering coefficient, characteristic path length and stability) calculated from EEG functional networks acquired when participants ($n=13$) are well rested (baseline). Furthermore, we investigated how stability (the consistency of a participant’s functional network over time measured using 2-D correlation) changed over the constant routine. We showed evidence of strong significant correlations between high mean node strength, low characteristic path length and high stability at baseline with a general resilience to extended sleep deprivation, although the same features lead to vulnerability during the period of natural sleep onset, highlighting non-uniform correlations over time. We also show significant differences in the levels of stability between resilient and vulnerable groups.

## Introduction

Sleep deprivation is endemic in society. A review of studies from the United Kingdom, the United States of America and the Netherlands showed that 1 in 4 people slept less than recommended for their age and 6.5% of adults slept less than 6 h^1^. Sleep deprivation can occur through sleep restriction or, as in this study, a total absence of sleep. In both cases sleep deprivation will enhance the homeostatic pressure for sleep^2^. The effects of sleep deprivation, and associated sleepiness, in humans are well documented^3^. Sleep deprivation has been associated, in part, with major human and environmental disasters^4^. This includes the Chernobyl nuclear reactor meltdown, the ill-judged launch of the fatal Challenger space shuttle and the 2013 Metro-North train crash^3,5^. In the US sleep deprivation was estimated to cost the economy $411 billion in 2015 due to higher levels of absenteeism (not attending work) or presenteeism (specifically attending work in a sub-optimal state for sleep related reasons)^6^. Sleep deprivation has a widespread impact on cognitive function, most typically captured through reaction times (RTs) on psychomotor vigilance tests (PVTs). General increases in RTs on PVTs as total time awake increases have been reproduced across many experimental protocols^7–10^. However, RTs are not simply a function of time awake but are controlled by the circadian rhythm of an individual. Indeed alertness levels or alternatively fatigue levels have generally thought to involve both homeostatic and circadian processes with the simplest models using a linear combination of the two as proxy^2,11^. The influence of circadian processes counteracting mounting sleep pressure results in relatively good performance during the biological day^12^, improved performance during the wake maintenance zone (WMZ)—a period that maximally promotes wakefulness before the release of melatonin^10^—and then a rapid deterioration during extended wakefulness^12^. However, despite these general trends participants sporadically achieve performance equivalent to their baseline even after total sleep deprivation, known as the state instability hypothesis^9^.

Furthermore, large individual differences in the response to sleep deprivation are observed. For example, Van Dongen et al.^8^ and Chua et al.^7^ both repeatedly sleep deprived participants and observed large inter-individual differences in behavioural outcomes, whilst intra-individual results were relatively stable. Similar inter-individual differences in vulnerability were seen in active-duty fighter pilots on a flight simulator after 38 h of sleep deprivation, highlighting systematic differences even between highly trained professionals replicating a real-world task^13^. It was hypothesised by Van Dongen et al. that the different responses to sleep deprivation were due to individuals having a specific trait, which controlled their vulnerability to sleep deprivation^8^.

This has naturally led to studies trying to establish a biomarker for an individual’s vulnerability to sleep deprivation. For example, Van Dongen et al.^8^ considered both prior sleep history and baseline performance, but neither explained the trait-like vulnerability. However, Chua et al. found that individual differences in baseline measures (encompassing the first 16 h) in PVT response, blink rate, heart rate, heart rate variability and electroencephalogram (EEG) spectral power in the alpha band were each able to explain at least 50% of the variability between participants. This suggests that trait-like differences to sleep deprivation occur in electrocardiogram and EEG measures in addition to behavioural outcomes.

EEG holds a wealth of information regarding brain activity, and network properties could impact the spread of perturbations like sleep deprivation. Nonetheless, to the best of our knowledge, functional networks (FNs) derived from EEG data have not been used to assess susceptibility to sleep deprivation, despite neuroimaging being a potential biomarker source^14^. EEG combined with functional connectivity and network analysis is a powerful non-invasive tool for analysing the complex system of the brain at the macroscale. Nodes in the network represent electrodes and the edges in the network represent the statistical dependencies^15^ of the brain signals recorded underneath each electrode. Analysing these dependencies could provide insights into how FN topology is associated with vulnerability to sleep deprivation. Indeed, it is plausible that the trait-like differences in the behavioural response to sleep deprivation could be underpinned by the topology of the individual’s FN, such that the robustness of this network to the perturbation of the circadian rhythm and sleep deprivation drives inter-individual differences in behavioural outcomes.

Therefore, in this study we use PVT performance from a 40 h constant routine (CR) to investigate if an individual’s response to sleep deprivation can be associated with information from their FNs obtained from well rested scalp EEG acquisitions before sleep deprivation. In particular, we consider the ability to correlate (i) an individual’s impairment (a singular metric of sleep deprivation effects) and (ii) an individual’s performance (continuous metrics across the CR) on a PVT, with graph metrics derived from FNs calculated using a baseline EEG acquisition. Furthermore, we use a stability analysis (the consistency of a participant’s FN over time measured using 2-D correlation) to further understand why PVT impairment differs between individuals.

## Methods

### Ethical approval

Ethical approval was provided by the Monash University Human Research Ethics Committee (CF14/2790-2014001546). All participants provided full, informed consent prior to the study, and the study was conducted in accordance with the Declaration of Helsinki. All provided data were already anonymised; therefore, no personal information could be associated with an individual.

### Participants and protocol

Inclusion criteria were (i) no artificial disruption to normal sleep patterns prior to the study (no shift work defined as 5 or more hours worked between 20:00 and 07:00 or transmeridian travel across three or more timezones) for at least the previous three months (ii) no prior diagnosis of medical, psychiatric or sleep disorders (iii) no extreme chronotype—the phase relationship between an individual’s circadian rhythm and the light-dark cycle of the sun (iv) fluency in English (v) a body mass index between 18.0 and 29.9 kg/m^2^ (vi) no reported use of illicit drugs in the previous year (vii) no consumption of caffeine exceeding 300 mg/day (viii) no alcohol consumption exceeding 14 standard unit/week (ix) non-smokers. Females in the study were not pregnant or using hormonal contraception and were studied in the laboratory during the follicular phase of their menstrual cycle. Before taking part in the study all participants underwent a medical and psychological screening by a physician and were deemed medically healthy based upon an electrocardiogram, blood and urine tests and an interview with a clinical psychologist. Furthermore, the experimental protocol ensured participants refrained from consuming alcohol and caffeine or using nicotine, supplements or prescription or non-prescription drugs for 3 weeks prior to the protocol beginning. These measures were verified through a urine toxicology screen and a breathalyser test at the being of the laboratory protocol.

Following participant recruitment 22 people were eligible and completed the protocol. However, data were missing from 9 subjects due to PVT technical difficulties. This resulted in the study having $n=13$ participants ($n=2$, female) $24.85 \pm 4.26$ years ($mean \pm std$) where PVT and EEG data were available. EEG from the 9 subjects were used to calculate baseline graph metrics, which were not dissimilar to those in the study ($p<0.05$, t-test) and the stability across the CR for these subjects is shown in the Supplementary Fig. S22.

These criteria ensured participants were not experiencing sleep disruption or circadian misalignment prior to the CR. Participants were good healthy sleepers, reporting habitual bedtimes between 21:30 and 01:00 and habitual wake times between 05:30 and 09:00. Sleep timings under habitual and structured sleep are available in Supplementary Table S1.

In addition, for the three weeks prior to laboratory settings and the first 2 days in the clinic a strict self-selected 8:16 h sleep:wake schedule was adhered to. Participants self-selected a bedtime between 22:00 and 01:00 such that they could sleep for 8 h while still being compatible with external obligations (e.g., work schedules). Our previous findings support the use of structured sleep schedules prior to in-laboratory sleep and circadian studies to stabilize sleep and circadian timing in healthy volunteers^16^ and avoids prior sleep deprivation influencing results. Indeed within the same participants the 8:16 schedule was found to stabilize sleep timing and improve the alignment of sleep onset and circadian phase while total sleep duration did not significantly differ compared to their habitual total sleep duration^16^. Compliance was checked via actigraphy data collected from Actiwatch Spectrum (Philips Repironics, BMedical, QLD, Australia), sleep diaries and time-stamped call-in messages upon waking and when participants went to bed. For a detailed description of the CR see McMahon et al.^10^.

**Fig. 1:**
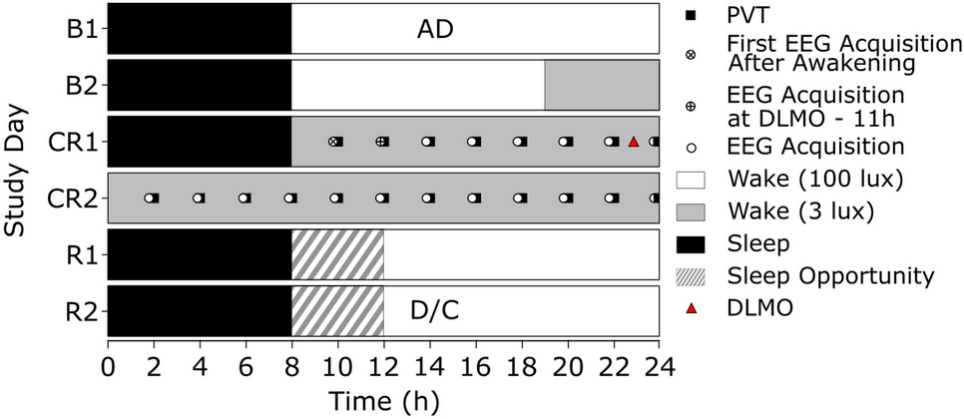
CR Protocol. Raster plot depicting the laboratory protocol across the 2 baseline days (B1, B2), the 2 days of CR (CR1, CR2) and 2 recovery days (R1, R2)—nominal 08:00 waketime and 23:00 DLMO as denoted by the red triangle. AD, admissions; D/C, discharge; circle EEG acquisition (120 s); circle and cross, first EEG acquisition after awakening (120 s); circle and plus, EEG acquisition at $\text {DLMO}-11\text { h}$ (120 s)—here DLMO was set at 23:00, as denoted by the red triangle, to provide an example of the first acquisition after awakening differing from the EEG acquisition at $\text {DLMO}-11\text { h}$.

The study consisted of a 6-day in lab protocol. After 2 days of acclimatisation within the laboratory (B1 and B2), participants awoke on the third day to a 40 h CR (CR1, CR2) followed by 2 days of recovery (12 h opportunity) sleep (R1, R2), as seen in Fig. 1. The CR consisted of 40 h continuous wake in private, sound-attenuated, temperature and light controlled suites. Participants were closely monitored to ensure sustained wakefulness and all time-cues were removed. Light ($\sim 3 \pm 1$ lux (horizontal) and $\sim 1 \pm 3$ lux (vertical)) and temperature levels (21 °C ± 2 °C) were kept constant. In addition, participants remained in a semi-recumbent position throughout. Finally, isocaloric snacks were provided hourly, following Australian Dietary Guidelines 2013 for macronutrients^17^.

### Dim light melatonin onset calculation

Melatonin levels were measured during the protocol for the calculation of Dim Light Melatonin Onset (DLMO) ($21:13 \pm 00:58$) to provide a marker of circadian phase per individual. Plasma melatonin levels for each participant were assessed through hourly blood samples starting approximately 1 h after starting CR. Values for hourly melatonin levels were then interpolated with DLMO times calculated as the first time that plasma melatonin concentration exceeded 5 pg/mL. Where possible DLMO was calculated for both the first and second evening of the CR. DLMO was only established using melatonin concentrations from the second evening when a recording for the first evening was not possible, for those with both nights the two nights were highly correlated $r=0.94$. The circadian phase for each participant was then set, such that DLMO on the first day was defined as time 0. Participant PVT data and EEG data were aligned by selecting the acquisition closest to *n* hours before or after that participant’s DLMO. For example, when aligned by DLMO the EEG acquisitions at $\text {DLMO}-11\text { h}$ took place $-11:05 \pm 00:39$ hours before DLMO while the PVT took place $-11:17 \pm 00:31$ hours before DLMO.

### Psychomotor vigilance test

Every 2 h each participant undertook a 10-min PVT. Here participants watched a white rectangle on the centre of the screen and had to respond with a thumb press as quickly as possible when the counter appeared. The stimulus for each trial appeared at a random interval of 2–10 s after the previous trial.

A RT under 100 ms was considered to be an anticipatory error and was removed from analysis in line with Basner and Dinges^18^. Trials with RT over 500 ms were labelled as lapses and were included in the analysis^18^. A RT over 5000 ms produced an alerting noise^10^ before the next trial started. The mean, median and std of RT, and the percentage of lapses from useable trials for each 10-min test, were calculated.

The results of certain testing sessions were not used in the analysis due to the test either not occurring or lasting less than the required 10 min ($n=13$ tests (5.26%) from $n=4$ participants). The available tests for each participant are given in Fig. S1.

### PVT impairment and performance

Performance was monitored throughout the CR using the PVT, where the mean, median and standard deviation of RT and percentage of lapses were recorded (Fig. 3 and Supplementary Fig. S3 display these time-series). Hence, for each participant we have up to 19 datapoints across the CR for PVT performance in a given metric.

We also wanted to detect impairment irrespective of when the worst performance occurred, removing inter-individual differences related to timing. For the mean, median and std of RT we calculated the magnitude of impairment as the percentage difference from the best performance prior to and include DLMO in CR1 (e.g., the biological day) to the worst performance post-DLMO (e.g., after the onset of sleep deprivation). For the percentage of lapses impairment was represented by their worst percentage of lapses post-DLMO. Hence, for each participant we have 1 value of PVT impairment in a given metric.

These time-periods were selected to separate the biological day from the onset of the biological night post-DLMO and hence the start of the extended sleep deprivation protocol. The timings of the PVT used for calculating impairment are presented in Supplementary Table S2 demonstrating that the timing of the best and worst performance can differ across both PVT metrics and participants.

The PVT impairment of each participant was then used to divide the participants into two groups. Hence, the 13 participants were split into the 6 lowest and 6 highest impaired participants for a given PVT metric, with 1 participant not included. The median participant was removed for the comparison of the impairment groups to allow greater differentiation between the high and low impairment groups and ensure equal group sizes, however they were included for the group and individual level analysis. Note that the participants were split by the level of impairment for each measure, hence the high and low impairment group changed per PVT metric.

### EEG acquisition

EEG data were recorded using 18 scalp electrodes from the standard 10-10 system (Fp1, Fp2, F3, F4, Fz, C3, C4, Cz, P3, P4, Pz, P7, P8, Po3, Po4, O1, O2, Oz). All EEG acquisitions were collected on a Compumedics Grael system with Profusion Sleep 4 software (Compumedics Ltd, Vic, Aus) with a sampling rate 512 Hz. EEG data were collected under Karolinska Drowsiness Test (KDT) conditions^19^. The KDT consisted of 3 min eye-open followed by 2 min of eye-closed and participants were instructed to remained relaxed and still. Due to the high alpha spectral power that occurs during well-rested eye-closed EEG^20^, only the eyes-closed acquisitions were utilised. This enabled the graph metric analysis to focus on the alpha frequency band. KDT conditions ensured a clear segment of wake EEG with minimal blink/movement artifact. This resulted in up to 19 acquisitions per participant. The first 2 min recording occurred 2 h after waking and subsequent recordings occurred every 2 h for a total of 40 h awake.

For acquisitions where one or more electrode time-series were corrupted, the entire acquisition was removed ($n=5$ acquisitions (2.02%) from $n=3$ participants covering $n=3$ electrodes). This was to ensure all EEG networks contained the same base network of 18 nodes making each FN directly comparable. In addition, $n=6$ (2.43%) EEG acquisitions from $n=3$ participants were missing, due to technical issues with the recording devices or participants requiring medical attention or experiencing distress. Supplementary Fig. S2 shows the available EEG recordings for each participant.

### EEG preprocessing

For each 2 min recording the EEG was referenced to the common average. The EEG acquisition was bandstop filtered between 49 and 51 Hz to remove powerline interference and filtered into the broadband (0.5–30 Hz). For the calculation of baseline graph metrics each epoch was filtered into the $8-14$ Hz (alpha) frequency band as resting state eyes closed EEG has high alpha spectral power when the participant is well rested^20^ and because Chua et al.^7^ found that only the alpha frequency band significantly explained intra-individual variance in task performance after sleep deprivation.

However, for the stability analysis four frequency bands were considered, $0.5-4$ Hz (delta), $4-8$ Hz (theta), $8-14$ Hz (alpha) and $14-30$ Hz (beta), chosen to ensure each frequency between 0.5 and 30 Hz was included. Stability was considered across all four frequency bands as the measure was calculated across the CR and the power spectral density of each frequency band is known to change with time awake. In particular alpha EEG power is known to decrease with time awake while theta EEG power increases^21,22^. Within each frequency band the data were then normalised such that each channel had mean 0 and standard deviation 1^23^.

### Baseline EEG acquisition

The baseline EEG acquisition was defined in two ways: $\hbox {B}_{{FA}}$ the first EEG acquisition after awakening (2 h post wake) and $\hbox {B}_{-11}$ the acquisition 11 h before DLMO (between 2 and 4 h post wake). These two baselines differ slightly by allowing an alignment based on time awake ($\hbox {B}_{{FA}}$) and circadian phase ($\hbox {B}_{-11}$). Note that while the calculation of circadian phase requires a participants to be under controlled settings for a minimum of 5 h^24^ time awake is trivial to establish. Therefore, all analyses were repeated for both baselines to investigate the need for circadian phase in future operational settings. For the majority of participants $\hbox {B}_{-11}$ was the same as $\hbox {B}_{{FA}}$ at $\sim 2$ h post wake, however for 3 participants their EEG acquisition 11 h before DLMO was acquired 4 h post wake.

### Functional network construction

For the baseline acquisition 30 s epochs were utilised and the 18 channels of filtered and normalised EEG data were used to calculate FNs. The phase locking factor (PLF) was used to calculate the functional connectivity between pairs of nodes^25^. Each frequency band filtered EEG timeseries was considered an oscillator, with the position of oscillator *j* at time $t \in \{1,\dots , L \}$ given by $z_j(t)=e^{i\theta _j(t)}$, where $\theta _j(t)$ is the oscillator’s phase. Then the level of synchronisation between pairs of nodes (e.g., nodes *j* and *k*) was summarised by how similar the phase of the two oscillators were,

1 $$\rho _{{j,k}} = \left| {\frac{1}{L}\sum\limits_{{t = 1}}^{L} {e^{{i(\theta _{k} (t) - \theta _{j} (t))}} } } \right|.$$

This resulted in undirected weighted adjacency matrices $\rho \in {\mathbb {R}}$, each with a size 18 $\times$ 18 and edge weights in the range [0, 1].

Surrogate analysis was then used to calculate the significance of each connection. 100 surrogate time-series were created by altering the bandpass filtered time-series using IAAFT—a Fourier-based method which ensures the autocorrelation and spectrum of the time-series were preserved^26^. The same methodology was used to create 100 surrogate PLF networks. Edges in the participant’s original PLF network remained if their weight was significantly higher than the corresponding edge from the surrogate networks at a 95% significance level, whilst non-significant edges were replaced by 0^27^.

In addition, the lag between each pair of EEG times series was calculated. Edges with a corresponding lag of zero were removed due to volume conduction^28^. Furthermore, the lag was used to make the surrogate-corrected PLF network directed by retaining edges with a positive lag^29^.

### Calculating graph metrics

Four graph metrics were then calculated for each alpha baseline PLF network: mean node strength, clustering coefficient, characteristic path length and stability.

**Fig. 2:**
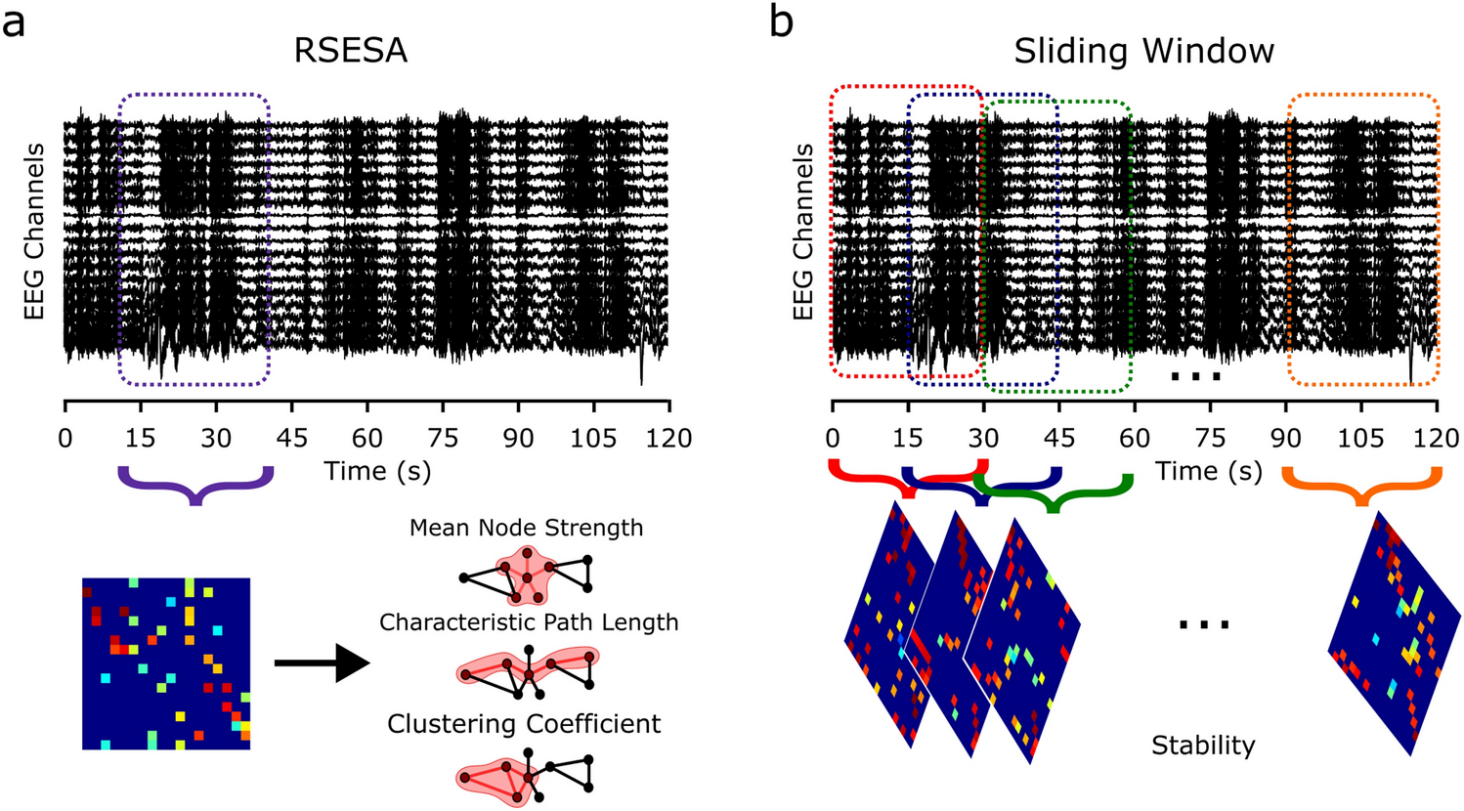
Epoch selection. (**a**) A single 30 s epoch selected using the Resting State Epoch Selector Algorithm, which was used for calculating a FN and the graph metrics mean node strength, characteristic path length and clustering coefficient. (**b**) The sliding window approach used to select 30 s epochs with a 15 s overlap. For each epoch a FN is calculated and the median 2-D correlation between each pair of FNs was taken as representative of stability.

For the mean node strength, clustering coefficient and characteristic path length analysis the FNs first underwent an order two edge reduction to remove connections where an alternative path via one or two additional nodes was stronger^27^, reducing the impact of indirect connections. Then the graph metrics were calculated using Brain Connectivity Toolbox^30^ for one 30 s epoch per baseline acquisition. Hence, a single 30 s epoch was selected from each baseline 120 s acquisition (Fig. 2a). EEG acquisitions were collected under supervision within a strict KDT protocol^19^. As a result, the artifacts were minimal and multiple 30 s epochs could have been selected. To provide an objective way to select an epoch a Resting State Epoch Selector Algorithm (RSESA) was used. The algorithm choose an epoch by finding 30 s of the acquisition which maximised the peak alpha frequency in the occipital electrodes O1 and O2, which is associated with expected eyes-closed restful EEG. Also, the algorithm minimised the variance of the power spectrum and minimised instantaneous correlation (zero lag correlation) over all electrodes to eliminate major artifacts.

The stability of an individual’s FN was defined to be the consistency of their PLF networks across all epochs in a given acquisition within a specific frequency band, as measured using 2-D correlation. This is based on network stability as presented and used by Shrey et al.^23^ and Smith et al.^31^. As multiple FNs were required for the correlation analysis the entire 120 s acquisition was split into 30 s epochs each overlapping their neighbour by half the epoch size (15 s) (Fig. 2b). This means all of the data were used and 7 epochs were created. For each of the 7 epochs, a PLF network was calculated. The stability of each EEG acquisition was then quantified by the 2-D correlation between each pair of the 7 PLF networks. Therefore, each PLF network was correlated to the other 6 PLF networks resulting in 21 correlations. The median stability was then taken as representative of the stability of the FNs for that EEG acquisition. The stability of the FNs was calculated across the 40 h CR for all four frequency bands.

### Statistical analysis

The PVT performance of the low and high impairment groups was compared using a non-parametric permutation test ($n=10,000$ permutations, two-tailed), establishing a *p*-value for the difference between the mean of the two groups for each measure over three equally sized ranges roughly split into biological day CR1 ($[\text {DLMO}-11\text { h}, \text {DLMO}]$)^32^, biological night ($[\text {DLMO}+1\text { h}, \text {DLMO}+12\text { h}]$) and biological day plus sleep deprivation ($[\text {DLMO}+13\text { h}, \text {DLMO}+24\text { h}]$). Grouping the timeseries by biological day and night was chosen to prevent the issue of multiple comparisons that could arise from comparing the groups across every time point while also allowing a direct comparison between the two biological days.

After the graph metrics were calculated from the baseline acquisitions, they were correlated to the level of PVT impairment using Pearson’s correlation. In addition, the Pearson’s correlation between the baseline graph metrics and PVT performance across the CR was calculated.

Within the stability analysis, increases in stability from prior to the WMZ to during the WMZ were assessed using a paired Cohen’s *d* test. The mean stability for each participant during the 3 h prior to the WMZ was compared to the 3 h during the WMZ. Normality of the data was determined through the Kolmogorov-Smirnov test. The associated *p*-values for both the group level and within high and low impairment groups are the results of associated paired two-tail *t*-tests. Furthermore, non-parametric permutation tests ($n=10,000$ permutations, two-tailed) were used to compare the stability of high and low impairment groups by comparing the mean for each participant across 12 h periods. Finally, the Spearman’s rank correlation between stability and std of RT from the PVT was calculated. At the individual level the timeseries for stability and the std of RT across the CR were normalised to have a mean of 0 and a standard deviation of 1, to ensure comparable scales.

Throughout the analysis the p-values were adjusted for multiple comparisons using the Benjamini-Hochberg method^33^ and $p<0.05$ was considered a significant result.

## Results

### Psychomotor vigilance test

**Fig. 3:**
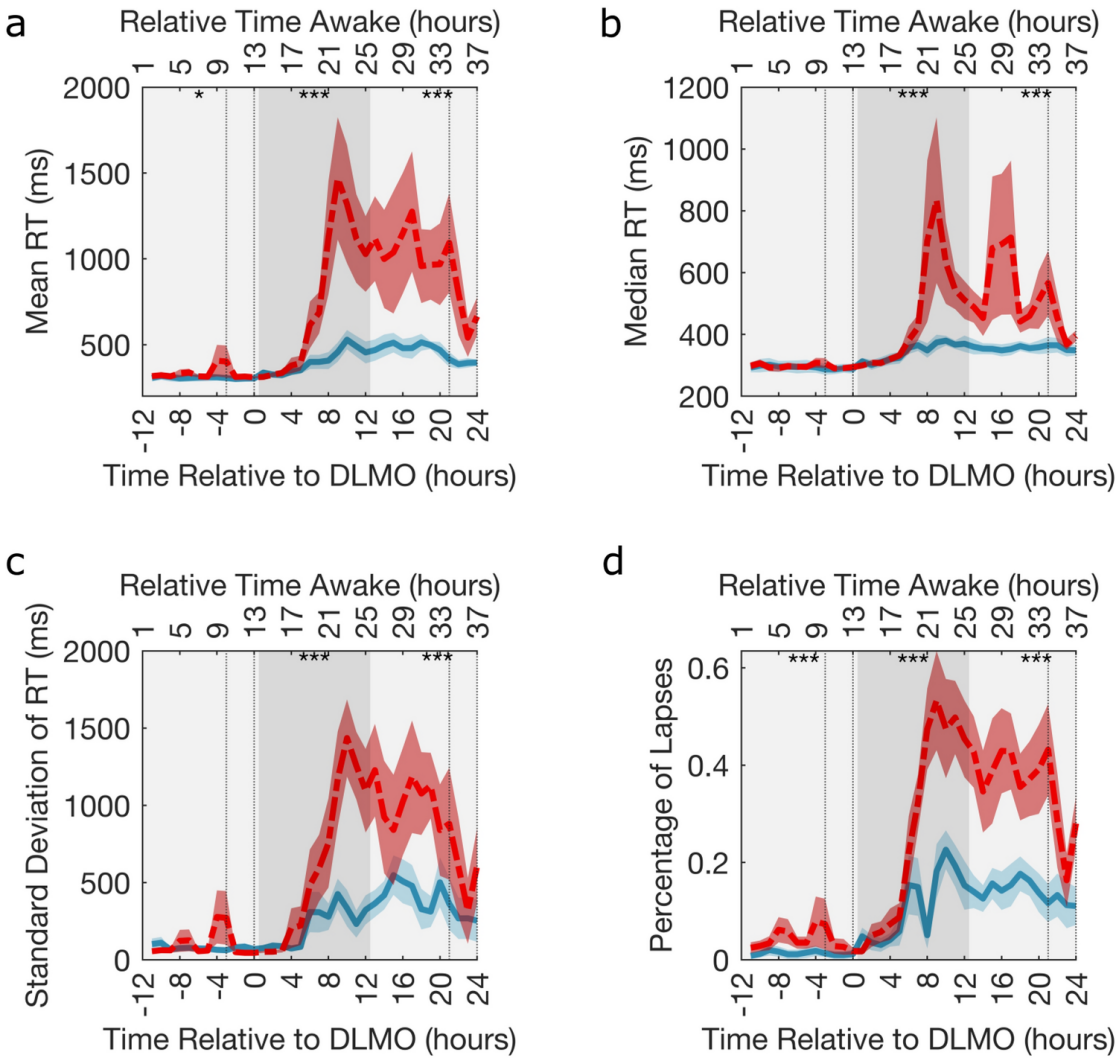
Psychomotor vigilance test performance split into groups based on high and low impairment. Timeseries showing (**a**) mean RT, (**b**) median RT, (**c**) standard deviation of RT and (**d**) the percentage of lapses when the participants are split into two groups based on the level of impairment in that measure. The solid blue line () and dashed red line () are the mean across the 6 lowest and 6 highest impaired participants, respectively. The corresponding blue and red shaded area is the standard error of the mean. The dotted vertical lines indicate the times considered to be in the WMZ (3 h before DLMO to 5 min after). The significance of permutation tests ($n=10,000$) comparing the mean value of the low and high impairment groups over adjacent 12-h periods depicted by different shades of grey are represented by, *$p<0.05$, **$p<0.005$, ***$p<0.0001$. In all plots the relative time awake provides an indication of the time awake for the majority of the participants.

The timeseries for each of the four PVT measures when the participants were split by low and high impairment are given in Fig. 3, corresponding group level figures are given in Supplementary Fig. S3. The general trend for all participants was stable good performance until $\sim \text {DLMO} +5 \text { h}$ when the PVT measures increase, reflecting an increase and greater variability in the RTs. The greater increase in the high impairment group is reflected by the results of the permutation tests ($p\le 0.0001$), which show a significant difference between the two impairment groups for all PVT measures when averaged over [$\text {DLMO}+1\text { h}$, $\text {DLMO}+12\text { h}$] and [$\text {DLMO}+13\text { h}$, $\text {DLMO}+24\text { h}$]. Also, a significant difference was found between the high and low impairment group for mean RT ($p=0.0130$) and percentage of PVT lapses ($p \le 0.0001$) when averaged over [$\text {DLMO}-11\text { h}$, $\text {DLMO}$].

Note that, a decrease in all measures, especially in the high impairment group, was seen around $\text {DLMO} +21 \text { h}$, with a local minimum occurring during the second WMZ, a time when the circadian rhythm maximally promotes wakefulness.

### PVT impairment

**Fig. 4:**
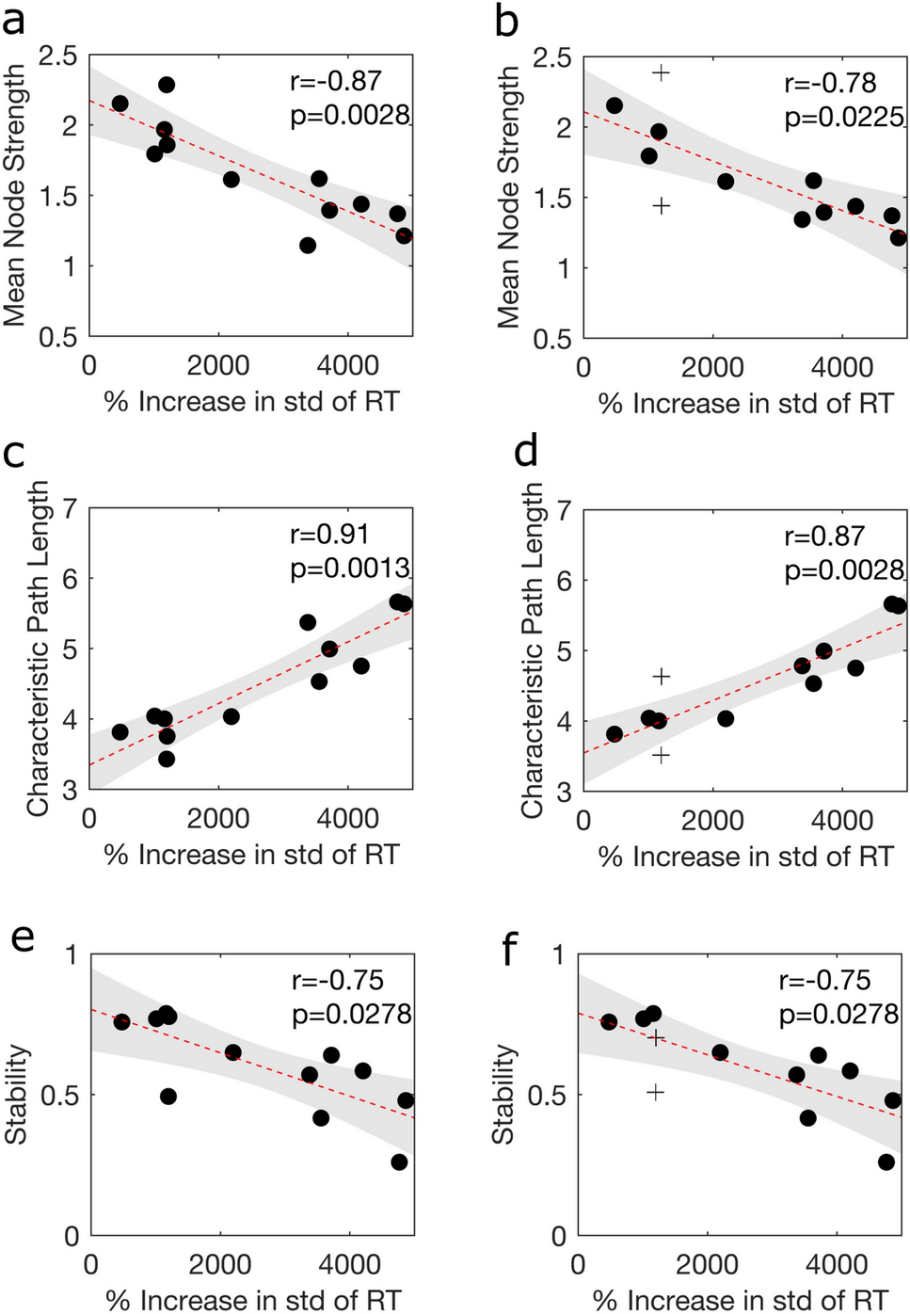
Significant relationships between PVT impairment and baseline graph metrics. Scatterplots relating the PVT impairment as measured using std of RT to (**a**) $\text {B}_\text {FA}$ mean node strength, (**b**) $\text {B}_\text {-11}$ mean node strength, (**c**) $\text {B}_\text {FA}$ characteristic path length, (**d**) $\text {B}_\text {-11}$ characteristic path length, (**e**) $\text {B}_\text {FA}$ stability and (**f**) $\text {B}_\text {-11}$ stability. For all plots, a black dot represents a participant, the red dashed line is the linear fit fitted using linear regression between the two variables and the grey patch represents the 95% confidence interval. In (**b, d, f**) the $+$ represents the participants whose acquisitions at $\text {DLMO}-11\text { h}$ differs from their first acquisition after awakening.

To assess if a baseline EEG acquisition provides information about the impairment of the participant under sleep deprivation the mean node strength, clustering coefficient, characteristic path length and stability of each baseline network were correlated to the level of impairment in each of the four PVT measures. The Pearson’s correlation and associated *p*-values are given in Supplementary Table S3. There were six metrics that significantly correlated to PVT impairment (Fig. 4). Indeed for $\text {B}_\text {FA}$ the std of RT had a significant correlation with mean node strength ($p=0.0028$), characteristic path length ($p=0.0013$) and stability ($p=0.0278$). Furthermore, for $\text {B}_\text {-11}$ the std of RT had a significant correlation with mean node strength ($p=0.0225$), characteristic path length ($p=0.0028$) and stability ($p=0.0278$). This indicates that networks with a high mean node strength, low characteristic path length and high stability are more robust to the effects of sleep deprivation as measured by std of RT. The figures for the other combinations are presented in the Supplementary Fig. S4 –S11.

Furthermore, to quantify the effect of female participants in our results, we repeated the analysis only considering male participants. The Pearson’s correlation were similar, albeit slightly reduced resulting in 3 significance relationships as seen in Supplementary Table S4.

For the median RT two participants were considered outliers, as seen in Supplementary Fig. S4b. The analysis was repeated without these participants, however their removal did not greatly change the correlations as seen in Supplementary Table S5.

### PVT performance

**Fig. 5:**
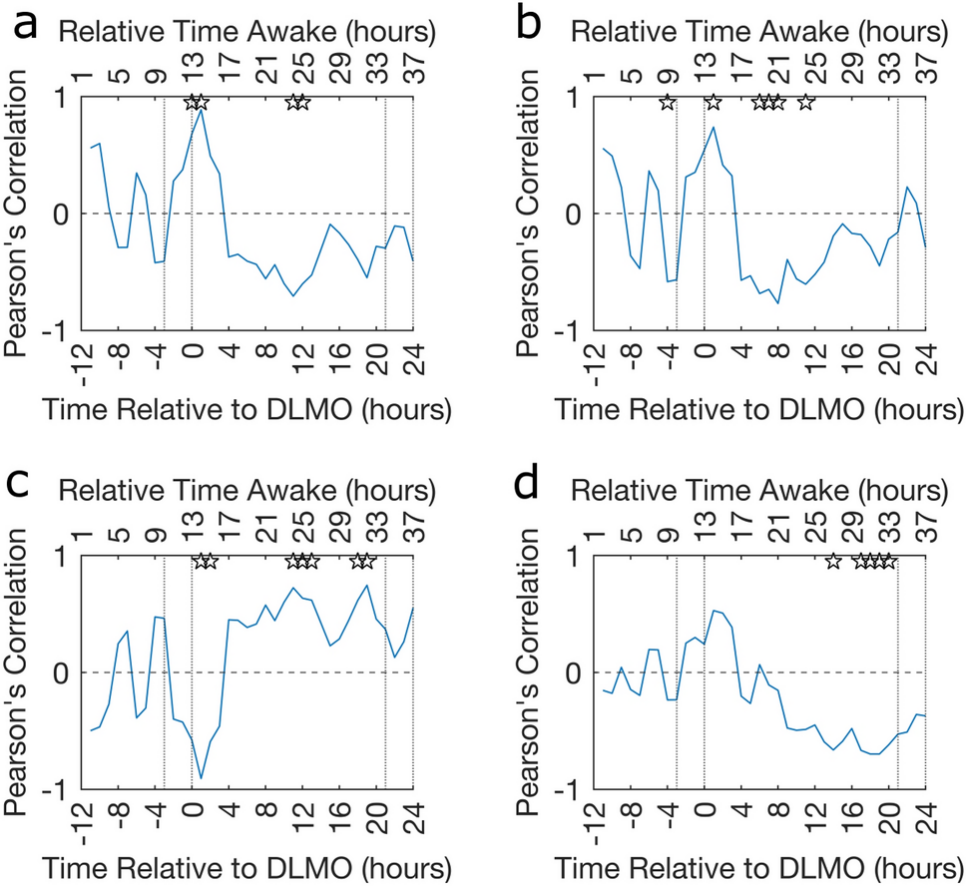
Pearson’s correlation between B$\mathbf {_{FA}}$ graph metrics and standard deviation of RT. How the Pearson’s correlation coefficient between baseline (**a**) mean node strength, (**b**) clustering coefficient, (**c**) characteristic path length and (**d**) stability for the first acquisition after awakening and the PVT performance as measured using std of RT. In all plots the ☆ indicates where the *p*-value associated with the correlation is significant $p<0.05$ (uncorrected) and the dotted vertical lines indicate the times considered to be in the WMZ (3 h before DLMO to 5 min after) and the relative time awake provides an indication of the time awake for the majority of the participants.

Within this section, the mean node strength, clustering coefficient, characteristic path length and stability of the baseline EEG acquisitions were correlated with PVT performance over the entire CR, rather than the single value used for PVT impairment. Note that positive correlations are indicative of high baseline graph metrics being associated with worse performance. The Pearson’s correlation between std of RT and the four graph metrics for $\text {B}_\text {FA}$ are presented in Fig. 5. Complimentary figures for $\hbox {B}_{-11}$ and the other PVT measures are in Supplementary Fig. S12–S20.

As seen in Fig. 5 the relationship between baseline graph metrics and PVT performance is more complex than the results presented from the PVT Impairment analysis suggest. There is a general trend that the correlation with maximum amplitude occurs at $\text {DLMO}+1\text { h}$ before correlations with a slightly reduced amplitude and opposite sign occur near $\text {DLMO}+11\text { h}$. This change in correlation sign indicates that at $\text {DLMO}+1\text { h}$ a high baseline mean node strength, clustering coefficient and stability as well as low characteristic path length are associated with poorer performance whilst near $\text {DLMO}+11\text { h}$ these baseline network markers are associated with better performance. This inverse relationship is shown clearly through scatterplots in Supplementary Fig. S13. However, while the trend is very clear and fairly consistent across the 2 baseline acquisitions, 4 graph metrics, 4 PVT performance metrics and 36 timepoints no significance remained after correcting the associated *p*-values for multiple comparisons.

### Stability

#### Group level

**Fig. 6:**
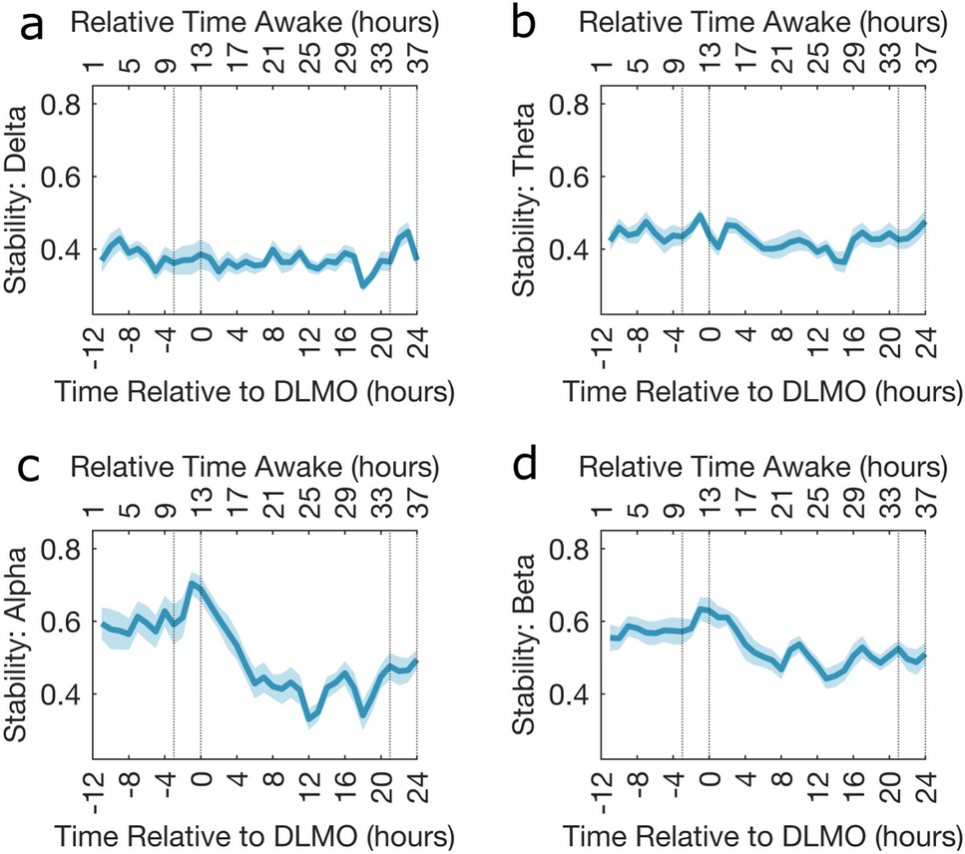
Stability of participant’s FNs. The stability across all participants for a given frequency band (**a**) Delta, (**b**) Theta, (**c**) Alpha and (**d**) Beta. The solid blue line is the mean across the participants stability and the shaded area is the standard error of the mean. In all plots the dotted vertical lines indicate the times considered to be in the WMZ (3 h before DLMO to 5 min after) and the relative time awake provides an indication of the time awake for the majority of the participants.

The stability of each participant for a given EEG acquisition within a frequency band was calculated and Fig. 6 shows the group level stability across participants.

Both alpha and beta frequency bands show consistent levels of stability during the first biological day before peaking in the WMZ. After the WMZ peak stability declined into the biological night before reaching a plateau, with some variation, during the second biological day. No visual trends were seen for delta or theta. Also, when comparing the stability prior to and during the first WMZ there were no significant differences for any frequency band as measured using permutation tests, as seen in Supplementary Table S6.

#### PVT groups

High and low impairment groups based on std of RT were utilised for the stability analysis, due to the observation that FNs were associated with this PVT measure in both the PVT Impairment and Performance analysis.

**Fig. 7:**
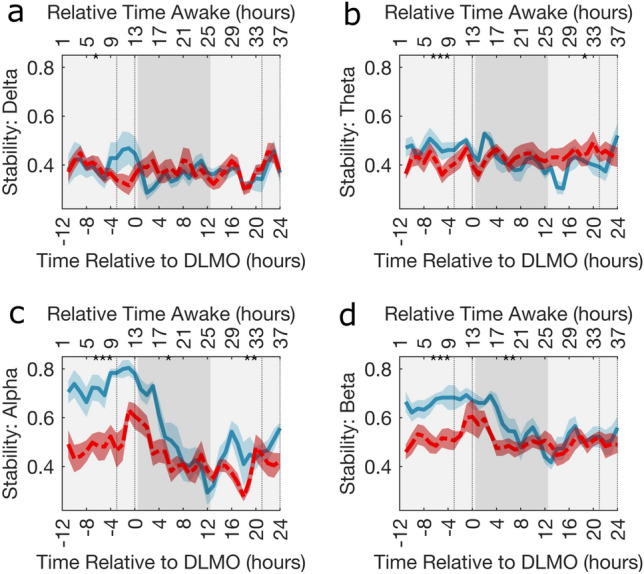
Stability of participant’s FNs with low and high impairment. The stability across the participants with the lowest PVT impairment and the highest PVT impairment as measured using the percentage increase in standard deviation of RT. The solid blue line () is the mean stability of the 6 participants with the lowest impairment and the dashed red line () is the mean stability of the 6 participants with the highest impairment. The corresponding blue and red shaded area is the standard error of the mean. Results are given for (**a**) Delta, (**b**) Theta, (**c**) Alpha and (**d**) Beta frequency bands. The significance of permutation tests ($n=10,000$) comparing the mean value of the low and high impairment groups over adjacent 12 h periods, depicted by different shades of grey, are represented by *$p<0.05$, **$p<0.005$, ***$p<0.0001$ (*p*-values provided in Supplementary Table S7). In all plots the dotted vertical lines indicate the times considered to be in the WMZ (3 h before DLMO to 5 min after) and the relative time awake provides an indication of the time awake for the majority of the participants.

As seen in Fig. 7 the two groups were significantly different during the first biological day in all frequency bands (see Supplementary Table S7 for *p*-values), the biological night in the alpha and beta bands and the second biological day in the theta and alpha bands.

**Table 1:** Stability prior to and during the WMZ for groups of high and low impairment.

	Low impairment			High impairment		
Frequency band	Paired Cohen’s *d*	*p*-value	Adjusted *p*-value	Paired Cohen’s *d*	*p*-value	Adjusted *p*-value
Delta	1.0548	0.0491	0.2386	0.2243	0.6063	0.7538
Theta	0.1214	0.6596	0.7538	0.6470	0.1739	0.3477
Alpha	0.3252	0.3644	0.5830	0.7327	0.1327	0.3477
Beta	0.0386	0.9521	0.9521	0.9906	0.0596	0.2386

Paired Cohen’s *d* and the associated *p*-values for paired *t*-tests comparing participants mean stability 3 h prior to the WMZ to the mean stability during the 3 h of the WMZ for a given frequency band within the high and low impairment group. Both uncorrected *p*-values and *p*-values corrected for multiple comparisons using the Benjamini-Hochberg method are provided^33^.

Indeed, in the alpha band, the low impairment group consistently had higher stability.

When comparing the stability prior to and during the first WMZ no significant differences occurred for any frequency band after correcting for multiple comparisons as seen in Table 1.

#### Individual level

To directly understand the relation between std of RT over the CR and the stability of the participant’s network these time-series were also compared using Spearman’s Rank correlation, this is displayed in Supplementary Fig. S21 and Supplementary Table S8.

Standard deviation of RT generally worsened over time and stability decreased, as summarised through the Spearman’s Rank Correlations of $-0.4737 \pm 0.2583$ ($mean \pm std$) and 9/13 participants having a significant inverse relationship. Indeed, only Participant 12 had a non-negative, albeit non-significant $(p\approx 1)$ correlation, likely due to their stability showing no noticeable drop below the first acquisition, unlike the other participants.

## Discussion

Within this study, we sought to understand the relationship between sleep deprivation and functional networks in the brain. In particular, we used only information from baseline EEG acquisitions to explore associations with robustness to sleep deprivation. Graph metrics from a baseline EEG acquisition were associated with the level of impairment, defined as the magnitude of sleep deprivation effects irrespective of time, on the PVT. High mean node strength, low characteristic path length and high stability at baseline significantly correlated with lower levels of impairment as measured using PVT (Fig. 4). This shows that a significant association between baseline FNs and task performance under sleep deprivation exists. A natural extension was to consider the association between baseline EEG acquisitions and PVT performance across the CR. This analysis showed that high mean node strength, high clustering coefficient, low characteristic path length and high stability at baseline were associated with more resilience to sleep deprivation during the second day of the CR, as measured using PVT RTs. Furthermore, these graph metric features were more strongly associated with reduced performance at $\text {DLMO}+1 \text { h}$ - when the drive to sleep starts to increase. Hence, in general the performance of the group decreased with time awake but within the group the ranking of the participants changed such that the individuals with the worst performance at $\text {DLMO}+1 \text { h}$ where among the best performers at $\text {DLMO}+11 \text { h}$, as seen in Supplementary Fig. S13.

The strength of the Pearson’s correlation when associating characteristic path length with both PVT impairment and performance demonstrated a similar trend to mean node strength, clustering coefficient and stability, however with the signs reversed. The association between high baseline mean node strength and better PVT performance after longer exposure to sleep deprivation is analogous to the results of Mu et al.^34^ and Caldwell et al.^35^. Both found associations between higher levels of global brain activity, as measured using fMRI, with resilience to the effects of sleep deprivation. The ratio of clustering coefficient and characteristic path length is commonly used in the calculation of the small-world index^36^; therefore combining the results for these graph metrics suggests that having a small-world network at baseline will result in more vulnerability to sleep deprivation immediately after DLMO—start of the biological night. However, a baseline small-world network would result in participants being more robust to sleep deprivation after being exposed for a longer period of time. Overall, this suggests that a more efficient and stable baseline network results in increased vulnerability to sleep near sleep onset and increased resilience to sleep near the end of the biological night. Potentially this could reflect the ability of the network to efficiently propagate the signals for sleep and wakefulness triggered by the circadian rhythm. Indeed computational models of small-world networks are commonly found to have enhanced signal-propagation speed^37^. How circadian signals for wakefulness at the macro-scale and the effect of wake promoting neurotransmitters at the mirco-scale interact is a topic for further research.

The std of RT negatively correlated with both baseline mean node strength and clustering coefficient near $[\text {DLMO}+8\text { h},\text {DLMO}+12\text { h}]$ and positively correlated with baseline characteristic path length near $\text {DLMO}+12\text { h}$ (Fig. 5). These correlations are consistent with the results from the PVT Impairment analysis, as seen in Fig. 4, although the magnitude was slightly lower for the performance analysis. Furthermore, the oppositely signed peak at $\text {DLMO}+1\text { h}$ was typically higher in amplitude than the Pearson’s correlations from the impairment analysis. Within this study we aligned the PVT data by DLMO and know that the drive to wakefulness in the WMZ and subsequent drive to sleep post DLMO is closely aligned across participants^10^. Indeed, we know that performance variability across participants is low during the WMZ and increases further away from DLMO^10^. Therefore, at $\text {DLMO}+1\text { h}$ less inter-individual differences in the timing of circadian and homeostatic process could have contributed to the greater effect size at $\text {DLMO}+1\text { h}$. Correspondingly, the increased variability in PVT performance across participants near $[\text {DLMO}+8\text { h},\text {DLMO}+12\text { h}]$ and intrinsic variability in the timing of each participants worst performance (Supplementary Table S2) could have contributed to the reduced effect size for the performance analysis compared to the impairment analysis.

Baseline EEG derived graph metrics do not correlate with PVT performance measures uniformly across the CR, with a switch occurring around DLMO into the biological night. As the baseline network for a participant remains constant this change in correlation is driven by changes in the participants’ PVT performance. Indeed, while sleep deprivation has a general negative effect on performance, the ranking of the participants almost reverses (Supplementary Fig. S13) such that the best individual for a task at $\text {DLMO}+1\text { h}$ would actually be among the worst to complete the task at $\text {DLMO}+11\text { h}$. Slight changes in the ranking over time would be expected due to inter-individual variability, however the reverse in the PVT performance ranking could be due to the influence of a participants’ circadian amplitude, such as the amplitude of core body temperature, metabolite rhythmicity or melatonin secretion^38,39^. This is because, if the monotonically increasing homeostatic process were the only influence, then the order of sleep deprivation susceptibility within the group would have no reason to reverse. However, a strong circadian amplitude that pushes an individual more towards sleep at night (worst performance) but also more towards alertness in the day (best performance) could explain reversed rankings and hence the change in correlation sign. We theorise that this change in the Pearson’s correlation sign could be a result of the amplitude of the circadian rhythm and hence the baseline graph metrics may be associated with circadian amplitude.

Across the impairment and performance analysis the Pearson’s correlations including std of RT were consistently strong. Indeed the std of RT was the only PVT impairment measure which was significantly correlated to baseline graph metrics (Fig. 4). The high correlations associated with std of RT could reflect the fact that the std of RT encompasses not just the number of lapses (percentage of lapses) or the magnitude of the lapse (mean RT and median RT) but rather a combination of both. Therefore, this PVT measure may be more effective at capturing both the sporadic good RTs and lapses that occur after extended sleep deprivation as theorised through the state instability hypothesis and hence why this PVT measure has the strongest correlations.

Across the PVT impairment and performance analysis all results were given for graph metrics aligned by circadian phase ($\hbox {B}_{-11}$) and time awake ($\hbox {B}_{{FA}}$). The results are largely similar, with $\hbox {B}_{{FA}}$ generally having a slightly reduced correlation in the impairment analysis for characteristic path length and stability but a general increase in correlation magnitude for mean node strength and clustering coefficient in the PVT performance analysis. By comparing the two baselines our results suggests that circadian phase was not a confounding factor in this analysis and that time awake, which is easier to calculate, is just as useful. Therefore, indicating that during future operational deployment of this framework time awake could be a sufficient. However, as participants were under structured sleep prior to the protocol and sleep disorders and extreme chronotypes were removed additional studies would be required to establish if time awake would continue to be useful.

Throughout this analysis the graph metrics were calculated using FNs created using the alpha frequency band. The alpha band was chosen due to the literature which shows the alpha band has the highest spectral content during the baseline acquisitions^40^. Also, Chua et al.^7^ found that only the alpha power spectrum could explain significant amounts of the variance between participants when they were sleep deprived. However, extending this research to include additional frequency bands could be of interest. Furthermore, while global graph metrics were considered in this study the level of correlation for individual electrodes could be considered in the future. For instance, Verweij et al.^41^ found that only scalp electrodes placed above the prefrontal and frontal brain regions were significantly impacted by sleep deprivation, therefore a focus on these electrodes could result in greater correlations.

Furthermore, motivated by the instability hypothesis^9^ the stability of the FNs across the CR was calculated, to understand how the brain reacts to sleep deprivation both in the long term over the protocol and the short term within an EEG acquisition. The stability across all participants in the alpha and beta frequency bands (Fig. 6) demonstrated an inverse relation to the PVT measures. Importantly, the stability of the FNs showed peaks during the WMZ, highlighting the effect of the circadian rhythm, while the drop in stability during the biological night shows the influence of the homeostatic pressure. This corresponds to the steep decrease in PVT performance during the biological night $\sim 5$ hours after DLMO before a marked improvement during the WMZ, as seen in Fig. S3. For both the PVT timeseries and the stability timeseries the influence of the homeostatic sleep pressure post DLMO seems to have the largest effect while the circadian influence is more subtle and focused on the WMZ. The similar relation between instability and PVT performance could provide evidence for the state instability hypothesis^9^, which suggests that brief returns to baseline levels occur alongside a general decrease in performance creating more variability. Indeed, instability and std of RT both increase with sleep deprivation reflecting increased variability in both FNs and behavioural performance, respectively.

When the participants were split into impairment groups, we saw a stark difference in their stability in all frequency bands during the first biological day, despite this being a time when neither group would have been experiencing sleep deprivation. When combined with the lack of significance difference between the high and low impairment groups for the median and std of RT (Fig. 7) this could suggest that the level of impairment a participant faces when sleep deprived is due to a mechanism that is present throughout the day, but only significantly affects performance when sleep debt accumulates. Interestingly, it is through sleep deprivation that the stability of the groups becomes more similar. This occurs due to the low impairment group decreasing their level of stability, rather than any marked change in the high impairment group. It is worth noting that the alpha band maintains a significant difference across the CR ($p \le 0.0161$). When combined with the PVT performances significantly differing ($p<0.0001$) across all four measures during the second and third 12 h of the protocol, Fig. 3, this could suggest that the stability in the alpha band is the most informative and most closely linked to PVT performance, as also indicated by the work of Chua et al.^7^.

Our results for stability could provide evidence to support a link between excitability and stability. For example, in the alpha and beta bands, especially for the low impairment group, stability generally decreases with increased time awake whilst excitability is known to increase with sleep deprivation^42,43^. Therefore, this suggests that high stability could be interpreted as less excitability and vice versa. Furthermore, we saw a visual increase in stability in the alpha and beta frequency bands as participants enter the first WMZ (Fig. 6)—especially in the high impairment group (Fig. 7) and this was also visually seen during the second WMZ for the low impairment group. Ly et al.^42^ found a reduction in cortical excitability from prior to during the WMZ, as measured by the significant decrease in the amplitude of a TMS-evoked EEG potential in the supplementary motor area. Again supporting the link between lower excitability and higher stability. Why the increase in stability during the WMZ is seen for different impairment groups in the two WMZs could be the focus of future studies.

At the individual level nine participants showed a significant association between stability of the baseline FNs and PVT performance (Supplementary Fig. S21). For instance, Participant 8 exhibited a clear inverse relationship between the stability of the network and the std of RT. The variability in the Spearman’s correlation between PVT performance and stability appears to be linked to the impairment groups as those that are the least impaired see a decrease in their stability over time, which more strongly corresponds to an increase in their std of RT. While for some participants a link between stability of FNs and the stability of their PVT RTs is seen the cause of this relationship remains unclear. However, it in unlikely that the stability of the FN directly impacts task performance, rather the same mechanism that impacts task performance also manifests as a less robust FN. For instance, prolonged wakefulness is known to increase the concentration of excitatory neurotransmitters^44^. Future research could investigate if there is an underlying link between neurotransmitters and the stability of FNs or alternatively PVT performance.

A natural extension of this research would be the prediction of individual susceptibility to sleep deprivation, using baseline graph metrics. Understanding susceptibility without requiring sleep deprivation could offer vital insights into the work patterns suitable for employees, especially in careers where acute sleep deprivation and sleep debt are likely and even unavoidable. For example, in the US 40.1% of healthcare professionals report getting insufficient sleep defined as less than 7 h of sleep while only 67% of military service members reported insufficient sleep, defined as $<7$ h for adults > 22 years and $<8$ h for adults < 21 years^45,46^. With further development, individualised prediction of sleep deprivation vulnerability could be used in a targeted manner within the employment process to help prioritise people for additional resources, mitigating their increased impairment before incidents occur.

In addition, throughout the correlation analysis at most two EEG acquisitions were considered per person. It is reasonable that other EEG acquisitions over the 40 h CR would have high levels of association and could be the focus of future research, especially as Chua et al.^7^ created one predictor using data across the first 16 h of wakefulness. However, our analysis could be seen as streamlining the approach of Chua et al.^7^ as high effect sizes ($r=0.89$) from a singular EEG acquisition were found, increasing practically for any real world application.

It is worth noting that, while significant correlations between baseline FNs and PVT performance have been found, an individual’s FN is not static and will change over time (Fig. 7). Indeed the presence of homeostatic and circadian rhythms in graph metric timeseries have been previously qualitatively noted within our stability analysis and in other sleep deprivation studies^47,48^, at the group level. Additionally, we have found significant associations between stability and PVT performance at the individual level. Further research is required to quantify the link between baseline graph metrics and the homeostatic and circadian processes that effect both PVT performance and graph metrics as sleep deprivation increases.

The results should be interpreted with the following limitations in mind. First, the PVT may not reflect actual vulnerability to sleep deprivation with respect to real-world operations, despite its utility in sleep deprivation research^18^. While it has been associated with memory^49^, lane position and speed variability in a simulated driving scenario^50^ as well as mathematical fluency and reading comprehension in children^51^ future work should examine how well PVT markers reflect performance in other tasks. Furthermore, to increase the relevance for real-world applications, this analysis framework could be applied to participants when they follow their habitual sleep patterns, since we have previously demonstrated the properties of functional brain networks are correlated with habitual sleep^52–54^. By focusing on habitual sleep more variation in sleep patterns would be possible as sleep disorders, shift work and extreme chronotype could be removed as exclusion criteria.

Second, for the PVT analysis the dataset included 13 participants, of which two were female, and thus we urge caution with the generalisabililty of the results. However, these 13 participants underwent a long and demanding protocol which resulted in large amounts of data from each individual, leading to significant correlations and differences. Indeed, our preliminary analysis has established associations between FNs and sleep deprivation and motivates further studies with additional participants.

Moreover, the low number of female participants will prevent generalising the results to female populations. To understand their impact on this study we repeated the PVT impairment analysis without any female participants and found only slight changes in the effect size (Supplementary Table S4). However, due to the clear sex difference in response to sleep loss^55–57^ this is an important area of future research. Beyond sex, different groups are known to have different response to sleep deprivation, as measured using PVT, including age^56^ and chronotype^52^. As the participants in this study were selected to create a homogeneous group, which is typical for sleep deprivation and biomarker development studies^58^, our findings motivate the use of our framework to investigate differences in the association of FNs and sleep deprivation across sex, age and chronotype differences.

Overall, we have completed a preliminary analysis into the association of FNs and performance impairment due to sleep deprivation. We have considered two angles of analysis: how are baseline FNs associated with the effect of sleep deprivation and how does the stability of the FNs help to understand the effect of sleep deprivation? We have seen that lower mean node strength, lower clustering coefficient, higher characteristic path length and less stability are features strongly associated with greater levels of impairment after a night of sleep deprivation, as measured using std of RT. However, these same features are associated with improved performance at $\text {DLMO}+1\text { h}$. Therefore, whilst the performance generally decreases throughout the CR the ranking of the participant’s performance almost reversed, suggesting the optimum person to complete a task at $\text {DLMO}+1\text { h}$ is actually amongst the worst at $\text {DLMO}+11\text { h}$, highlighting the time dependent nature of this analysis. The topology of the FNs could be related to a combination of circadian amplitude and the ability to propagate signals related to the circadian rhythm. Also, the stability of the network may help to explain why some participants are more resilient to the effects of sleep deprivation and why PVT performance generally decreases with wakefulness.

## Supplementary Information

Supplementary Information.

## Data Availability

The datasets analyzed during the current study are available upon reasonable request by contacting S.L.M.
