# Supplementary material for: Associating EEG functional networks and the effect of sleep deprivation as measured using psychomotor vigilance tests
Source: Sci Rep. 2024 Nov 14;14:27999. doi: 10.1038/s41598-024-78814-4 (PMC11564749; doi:10.1038/s41598-024-78814-4)
Supplement: Supplementary file 1 — Supplementary Information. [file 41598_2024_78814_MOESM1_ESM.pdf]

Associating EEG Functional Networks and the Effect of Sleep Deprivation as Measured Using Psychomotor Vigilance Tests

Sophie L. Mason[1,2*], Leandro Junges[1], Wessel Woldman[1,3], Suzanne Ftouni[4], Clare Anderson[2,4], John R. Terry[1,3,+], and Andrew P. Bagshaw[2,+]

[1]University of Birmingham, Centre for Systems Modelling and Quantitative Biomedicine, Birmingham, B15 2TT, United Kingdom
[2]University of Birmingham, Centre for Human Brain Health, College of Life and Environmental Sciences, Birmingham, B15 2TT, United Kingdom
[3]Neuronostics Limited, Engine Shed, Station Approach, Bristol, United Kingdom
[4]Monash University, Turner Institute for Brain and Mental Health, School of Psychological Sciences, Clayton, VIC, 3800, Australia
[*]s.l.mason@bham.ac.uk
[+]these authors contributed equally to this work as senior author

S1 Habitual and Structured Sleep Times

The participants' sleep timings were monitored for 3 weeks prior to the laboratory protocol for full details see McMahon et al.[1]. The first week involved unstructured sleep, allowing participants to follow their habitual sleep timings. For the following two weeks the participants followed a strict 8:16 sleep:wake schedule. The average timings for bedtime, waketime and total sleep time (TST) were monitored using actigraphy and sleep diaries these are given in Supplementary Table S1.

Participant	Sex	Bedtime		Waketime		TST (h)	
		Habitual	Structured	Habitual	Structured	Habitual	Structured
1	M		23:30 ± 00:00		07:30 ± 00:00		8 ± 0
2	F	00:59 ± 00:49	23:38 ± 00:04	09:08 ± 01:15	07:30 ± 00:00	7.86 ± 1.97	7.61 ± 0.31
3	M	22:45 ± 00:29	23:00 ± 00:00	07:31 ± 00:30	06:59 ± 00:00	8.47 ± 0.43	7.54 ± 0.32
4	F		23:12 ± 00:47		06:57 ± 00:19		7.75 ± 0.53
5	M	23:30 ± 00:28	23:15 ± 00:32	07:24 ± 00:47	07:09 ± 00:36	7.71 ± 0.47	7.70 ± 0.34
6	M	22:30 ±00:30	22:00 ± 00:00	06:20 ± 00:40	06:01 ± 00:05	7.25 ± 0.42	7.20 ± 0.25
7	M	22:07 ± 00:24	23:00 ± 00:00	07:32 ± 00:51	06:49 ± 00:22	8.91 ± 0.97	7.55 ± 0.53
8	M	00:34 ± 00:45	23:40 ± 00:01	07:45 ± 00:24	07:40 ± 00:00	6.81 ± 0.85	7.60 ± 0.20
9	M	00:56 ± 00:57	23:31 ± 00:09	05:14 ± 08:07	07:30 ± 00:00	8.23 ± 1.56	7.87 ±0.13
10	M	00:16 ± 01:24	23:00 ± 00:01	08:56 ± 01:13	07:00 ± 00:20	7.99 ± 0.94	7.60 ± 0.44
11	M	22:30 ± 00:00	22:30 ± 00:01	05:55 ± 00:23	06:07 ± 00:28	6.98 ± 0.66	7.25 ± 0.50
12	M	23:59 ± 00:57	23:30 ± 00:02	08:34 ± 00:16	07:30 ± 00:00	8.18 ± 0.84	7.62 ± 0.20
13	M	00:06 ± 00:36	22:30 ± 00:00	08:23 ± 01:26	06:30 ± 00:00	8.11 ± 1.14	7.67 ± 0.24

Table S1. Habitual and Structured Sleep Timings. The mean and standard deviation of each participant's bedtime, waketime and total sleep time (TST) over 1 week of habitual (unstructured) sleep and two weeks of structured sleep. Actigraphy data is provided expect for Participants 4, 9, 13 where the sleep diary due to technical difficulties with the watches. Participants 1 and 4 did not complete the habitual sleep monitoring.

Data Available

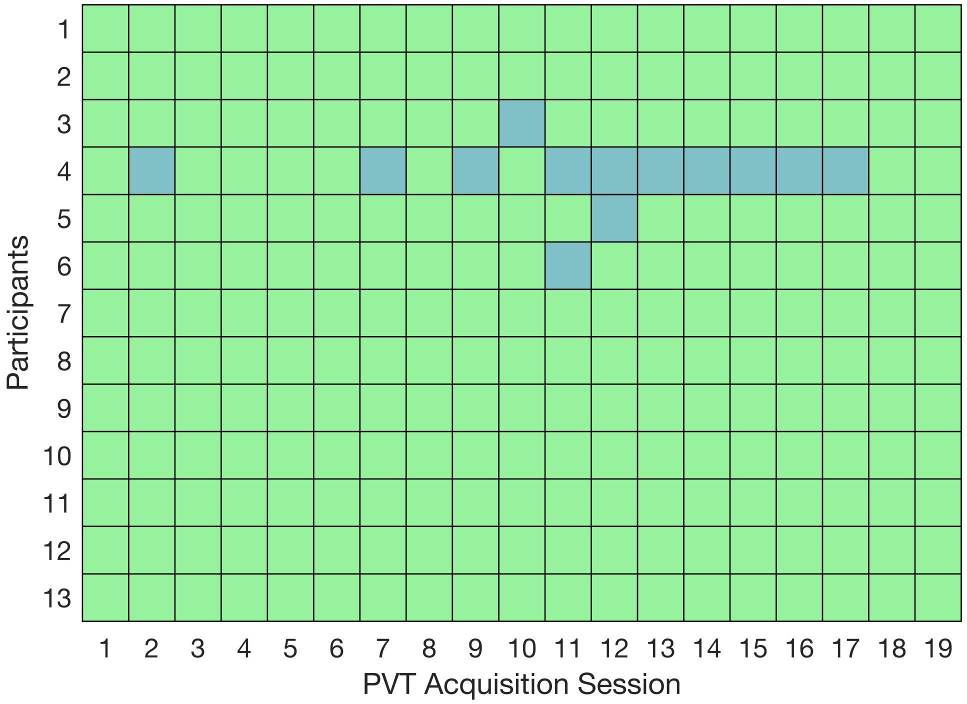

Figure S1. PVT Acquisitions Available. The available PVT acquisition sessions for each participant across the CR. The colours represent [green] a successful PVT acquisition and [blue] unable to record a PVT acquisition.

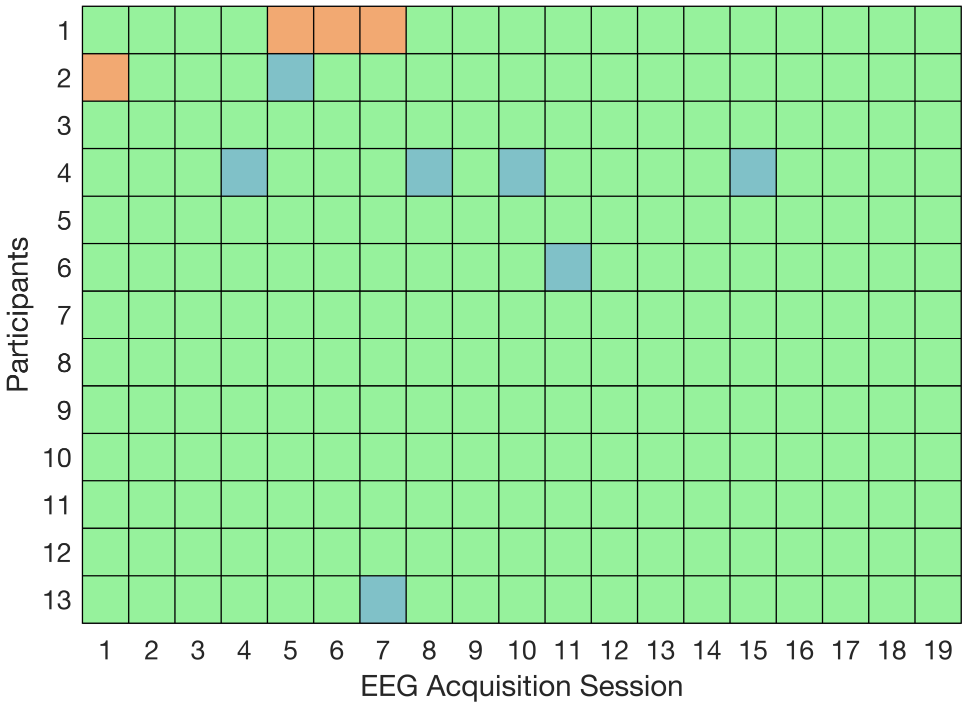

Figure S2. EEG Acquisitions Available. The available EEG acquisition sessions for each participant across the CR. The colours represent a successful EEG acquisition, an EEG acquisition with at least one faulty electrode and unable to record an EEG acquisition.

PVT Impairment and Performance

Participant	Mean RT (ms)		Median RT (ms)		Standard Deviation of RT (ms)		Percentage of Lapses	
1	-1,0	17,18	-11,-10	9,10	-3,-2	15,16	-	9,10
2	-2,-1	14,15	0	20,21	0	14,15	-	20,21
3	-2,-1	10,11	-2,-1	10,11	-2,-1	12,13	-	10,11
4	-4,-3	6,7	-4,-3	6,7	-4,-3	6,7	-	6,7
5	-11	20,21	-6,-5	20,21	0	20,21	-	20,21
6	-3,-2	9,10	-3,-2	6,7	-1,0	9,10	-	6,7
7	-9,-8	19,20	-9,-8	23,24	-9,-8	19,20	-	23,24
8	-10,-9	10,11	-11	10,11	-10,-9	10,11	-	10,11
9	-1,0	9,10	-1,0	17	-1,0	9,10	-	15,16
10	-2,-1	8,9	-2,-1	8,9	0	16,17	-	8,9
11	-7,-6	11,12	-7,-6	21,22	-7,-6	11,12	-	11,12
12	-9,-8	17,18	-9,-8	19,20	-9,-8	17,18	-	19,20
13	-2	10,11	-6,-5	10,11	-1,0	10,11	-	10,11

Table S2. Timings of the Minimum and Maximum PVT Performance with Respect to DLMO. For each of the four PVT performance measures the timing of the minimum mean RT, median RT, standard deviation of RT and percentage of lapses with respect to DLMO is given in the first column and the maximum is given in the second column. The timing for the minimum percentage of lapses is not provided as this is not used when assessing impairment.

PVT Group Level

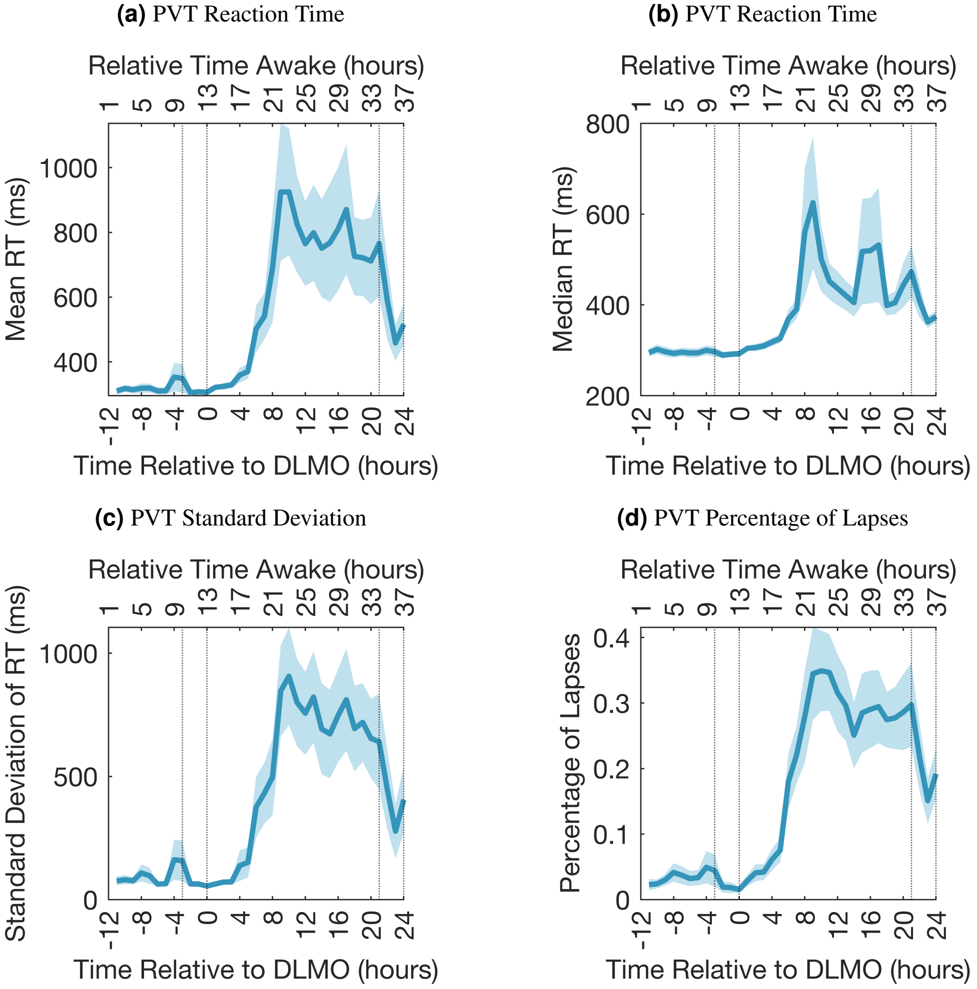

Figure S3. Psychomotor Vigilance Test Performance. The performance of all participants in the PVT as measured using **(a)** mean RT, **(b)** median RT, **(c)** standard deviation of RT and **(d)** percentage of lapses at each PVT session, relative to DLMO (time zero). The solid blue line is the mean across the participants' PVT metric and the shaded area is the standard error of the mean. The dotted vertical lines indicate the times considered to be in the WMZ (3 hours before DLMO to 5 minutes after) and the relative time awake provides an indication of the time awake for the majority of the participants.

PVT Impairment

Baseline Acquisition: Graph Metric: PVT Impairment	Correlation	p-value	Adjusted p-value
B_{FA}: $\langle s \rangle$: Mean RT	-0.5618	0.0573	0.1411
B_{FA}: $\langle s \rangle$: Median RT	-0.3538	0.2592	0.2799
B_{FA}: $\langle s \rangle$: std RT	-0.8667	0.0003	0.0028*
B_{FA}: $\langle s \rangle$: % Lapses	-0.3748	0.2299	0.2799
B_{FA}: $\langle C \rangle$: Mean RT	-0.4315	0.1613	0.2347
B_{FA}: $\langle C \rangle$: Median RT	-0.3669	0.2407	0.2799
B_{FA}: $\langle C \rangle$: std RT	-0.6078	0.0360	0.1048
B_{FA}: $\langle C \rangle$: % Lapses	-0.3516	0.2624	0.2799
B_{FA} :λ_L: Mean RT	0.6199	0.0315	0.1016
B_{FA} :λ_L: Median RT	0.4171	0.1773	0.2467
B_{FA} :λ_L: std RT	0.9093	<0.0001	0.0013*
B_{FA} :λ_L: % Lapses	0.4605	0.1320	0.2347
B_{FA}: r_s: Mean RT	-0.6193	0.0317	0.1016
B_{FA}: r_s: Median RT	-0.4497	0.1425	0.2347
B_{FA}: r_s: std RT	-0.7474	0.0052	0.0278*
B_{FA}: r_s: % Lapses	-0.4434	0.1488	0.2347
$B_{\text{-}11}$: $\langle s \rangle$: Mean RT	-0.5490	0.0645	0.1475
$B_{\text{-}11}$: $\langle s \rangle$: Median RT	-0.3520	0.2618	0.2799
$B_{\text{-}11}$: $\langle s \rangle$: std RT	-0.7793	0.0028	0.0225*
$B_{\text{-}11}$: $\langle s \rangle$: % Lapses	-0.4327	0.1600	0.2347
$B_{\text{-}11}$: $\langle C \rangle$: Mean RT	-0.3419	0.2768	0.2857
$B_{\text{-}11}$: $\langle C \rangle$: Median RT	-0.2817	0.3750	0.3750
$B_{\text{-}11}$: $\langle C \rangle$: std RT	-0.4085	0.1874	0.2499
$B_{\text{-}11}$: $\langle C \rangle$: % Lapses	-0.3524	0.2612	0.2799
$B_{\text{-}11}$:λ_L: Mean RT	0.6566	0.0204	0.0931
$B_{\text{-}11}$:λ_L: Median RT	0.4563	0.1360	0.2347
$B_{\text{-}11}$:λ_L: std RT	0.8750	0.0002	0.0028*
$B_{\text{-}11}$:λ_L: % Lapses	0.5741	0.0510	0.1359
$B_{\text{-}11}$: r_s: Mean RT	-0.6341	0.0268	0.1016
$B_{\text{-}11}$: r_s: Median RT	-0.4547	0.1375	0.2347
$B_{\text{-}11}$: r_s: std RT	-0.7488	0.0051	0.0278*
$B_{\text{-}11}$: r_s: % Lapses	-0.4671	0.1257	0.2347

Table S3. Results of correlating the Baseline Graph Metric with PVT Impairment. The Pearson's correlation between the participants' PVT impairment and the graph metric for both baseline acquisitions when correlating mean node strength ($\langle s \rangle$), clustering coefficient ($\langle C \rangle$), characteristic path length (λ_L) and stability (r_s) with the four PVT measures. The p-values were corrected for multiple comparisons using the Benjamini-Hochberg correction for FDR[2]. * denotes significance p-value < 0.05.

Mean Node Strength

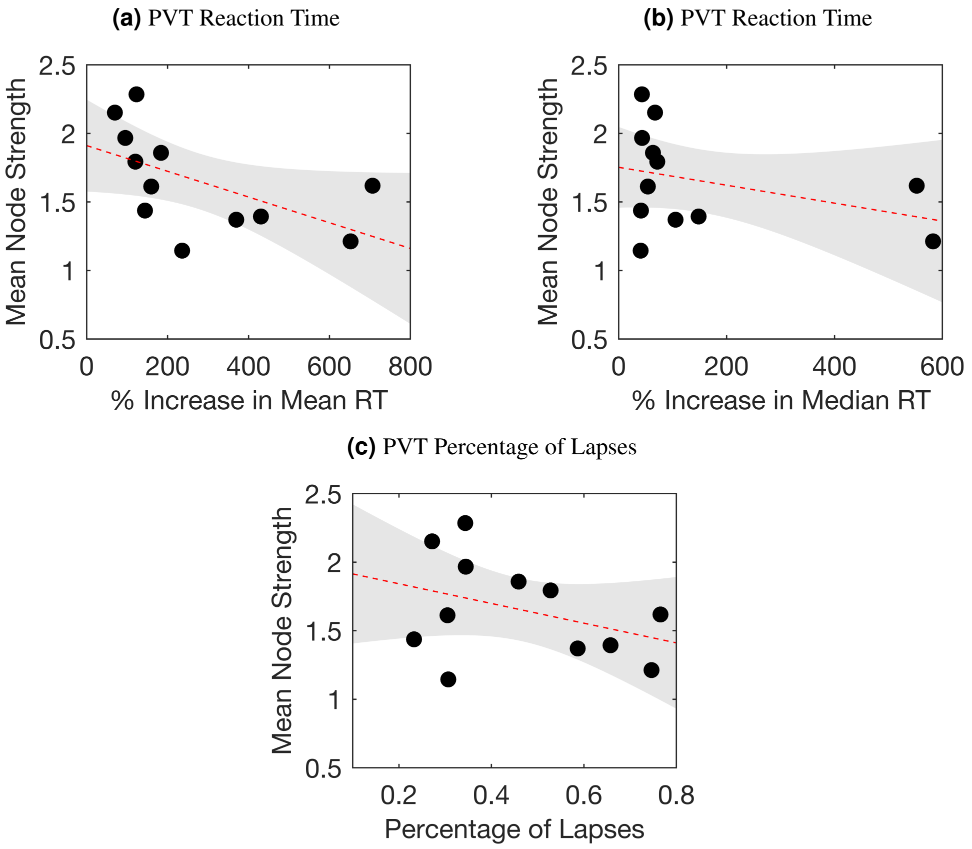

Figure S4. Scatterplot Relating PVT Impairment to B_{FA} Mean Node Strength. Scatterplots relating the PVT impairment as measured using **(a)** mean RT, **(b)** median RT and **(c)** percentage of lapses to mean node strength for the first acquisition after awakening. For all plots, a black dot represents a participant, the red dashed line is the linear fit fitted using linear regression between the two variables and the grey patch represents the 95% confidence interval.

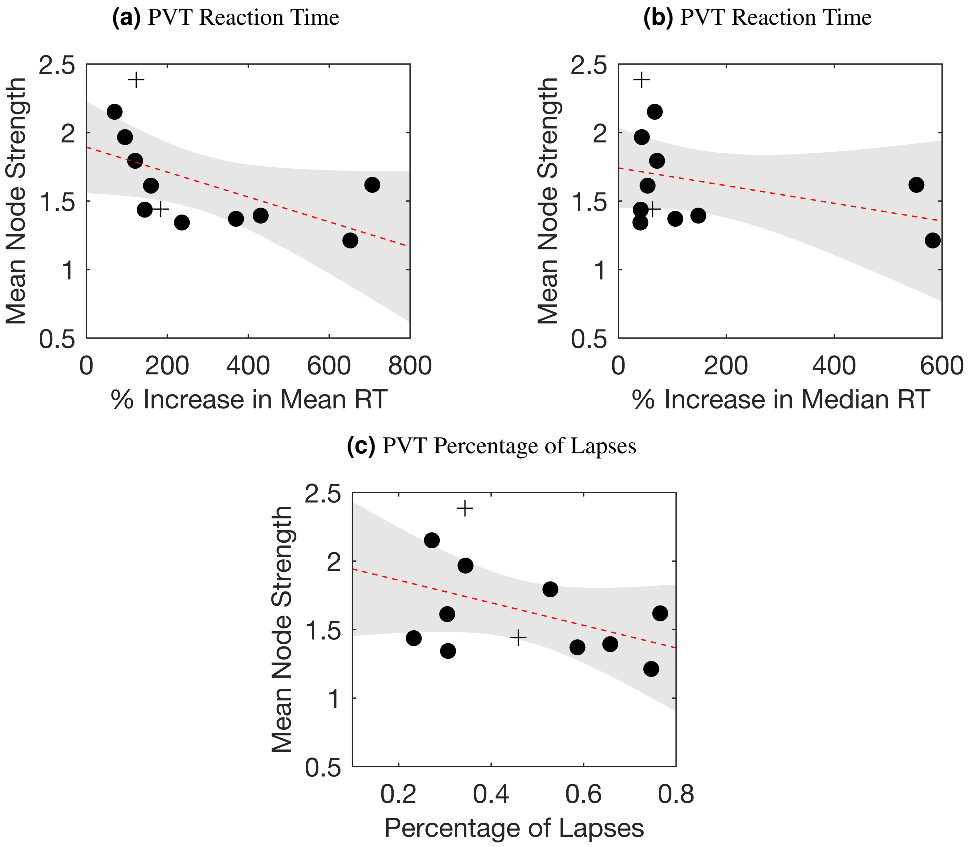

Figure S5. Scatterplot Relating PVT Impairment to B_{-11} Mean Node Strength. Scatterplots relating the PVT impairment as measured using **(a)** mean RT, **(b)** median RT and **(c)** percentage of lapses to mean node strength for the acquisition 11 hours before DLMO. For all plots, participants whose EEG acquisition 11 hours before DLMO is not their first recording are represented by $+$, while those whose first EEG acquisition is 11 hours before DLMO are denoted by • a black dot, the red dashed line is the linear fit fitted using linear regression between the two variables and the grey patch represents the 95% confidence interval.

Clustering Coefficient

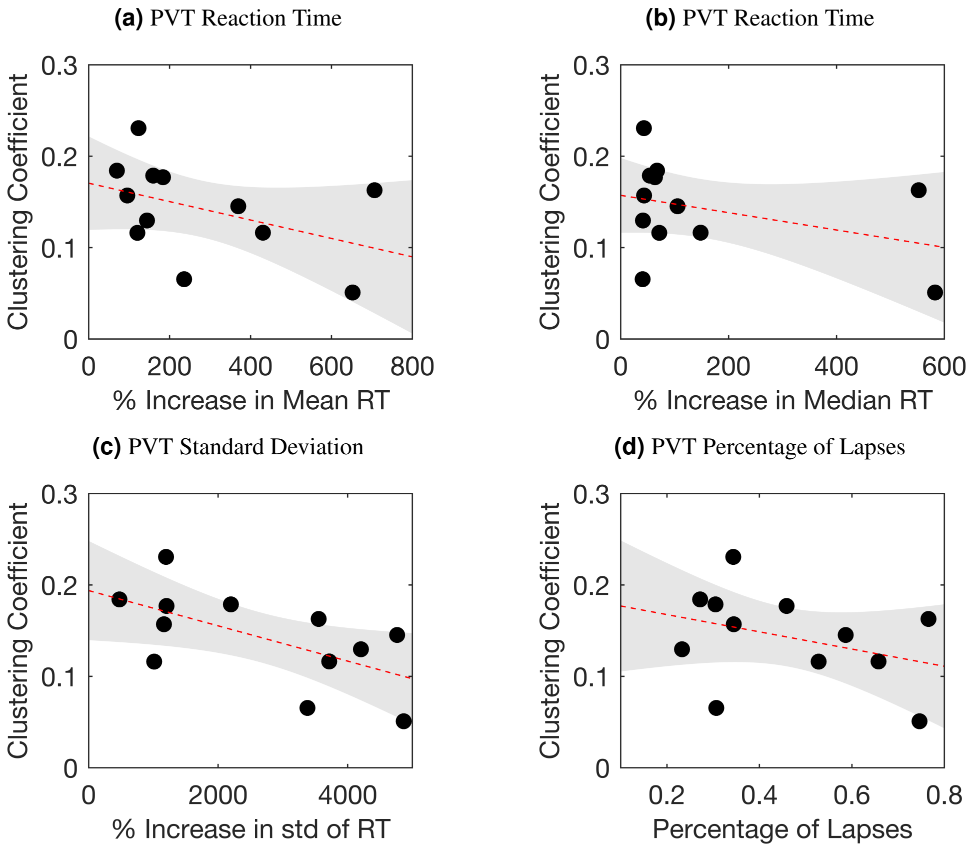

Figure S6. Scatterplot Relating PVT Impairment to B_{FA} Clustering Coefficient. Scatterplots relating the PVT impairment as measured using **(a)** mean RT, **(b)** median RT, **(c)** standard deviation of RT and **(d)** percentage of lapses to the clustering coefficient for the first acquisition after awakening. For all plots, a black dot represents a participant, the red dashed line is the linear fit fitted using linear regression between the two variables and the grey patch represents the 95% confidence interval.

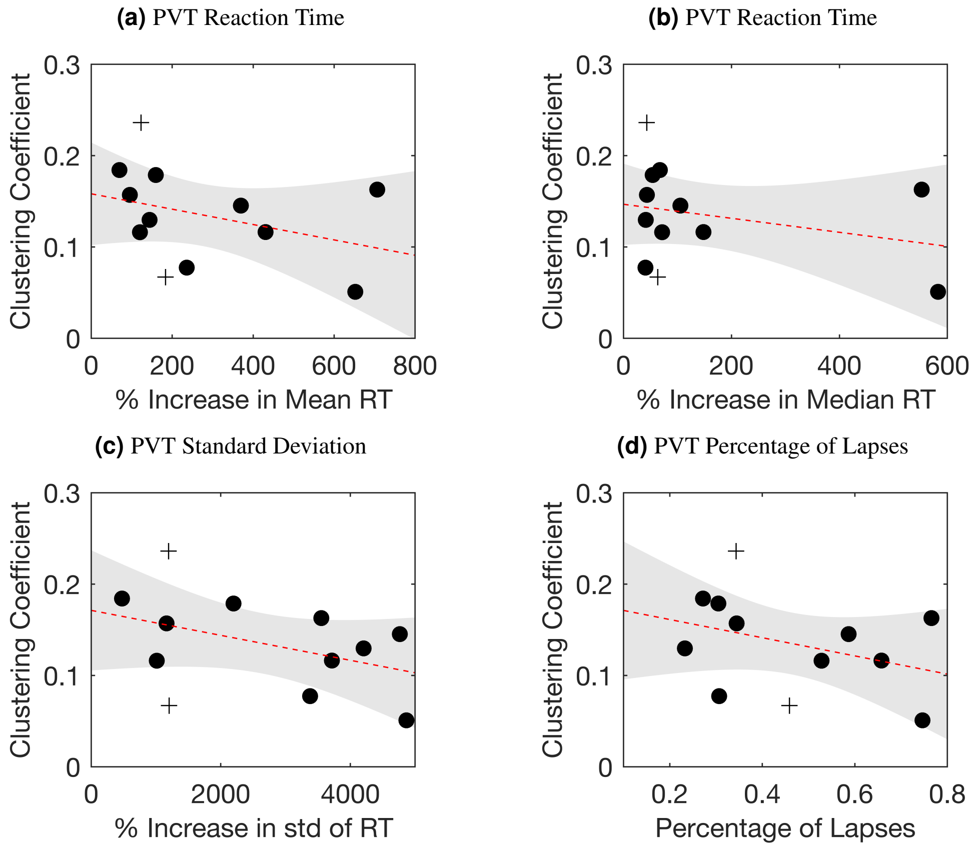

Figure S7. Scatterplot Relating PVT Impairment to B_{-11} Clustering Coefficient. Scatterplots relating the PVT impairment as measured using **(a)** mean RT, **(b)** median RT, **(c)** standard deviation of RT and **(d)** percentage of lapses to the clustering coefficient for the acquisition 11 hours before DLMO. For all plots, participants whose EEG acquisition 11 hours before DLMO is not their first recording are represented by +, while those whose first EEG acquisition is 11 hours before DLMO are denoted by • a black dot, the red dashed line is the linear fit fitted using linear regression between the two variables and the grey patch represents the 95% confidence interval.

Characteristic Path Length

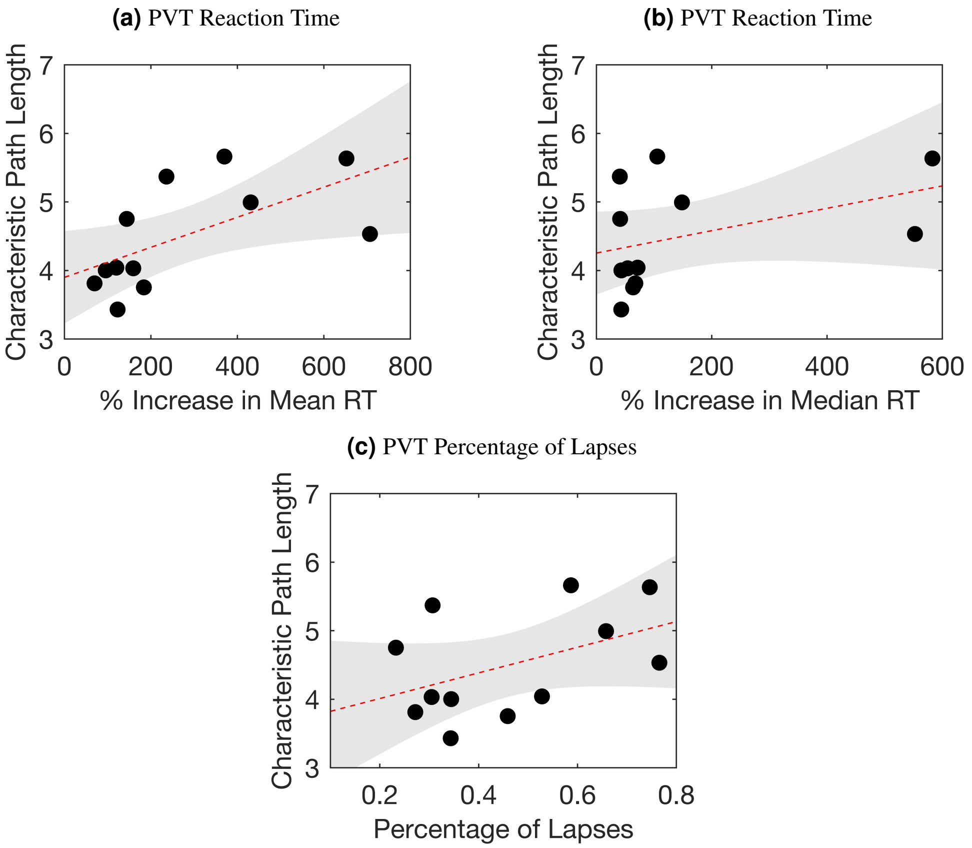

Figure S8. **Scatterplot Relating PVT Impairment to B_{FA} Characteristic Path Length.** Scatterplots relating the PVT impairment as measured using **(a)** mean RT, **(b)** median RT and **(c)** percentage of lapses to the characteristic path length for the first acquisition after awakening. For all plots, a black dot represents a participant, the red dashed line is the linear fit fitted using linear regression between the two variables and the grey patch represents the 95% confidence interval.

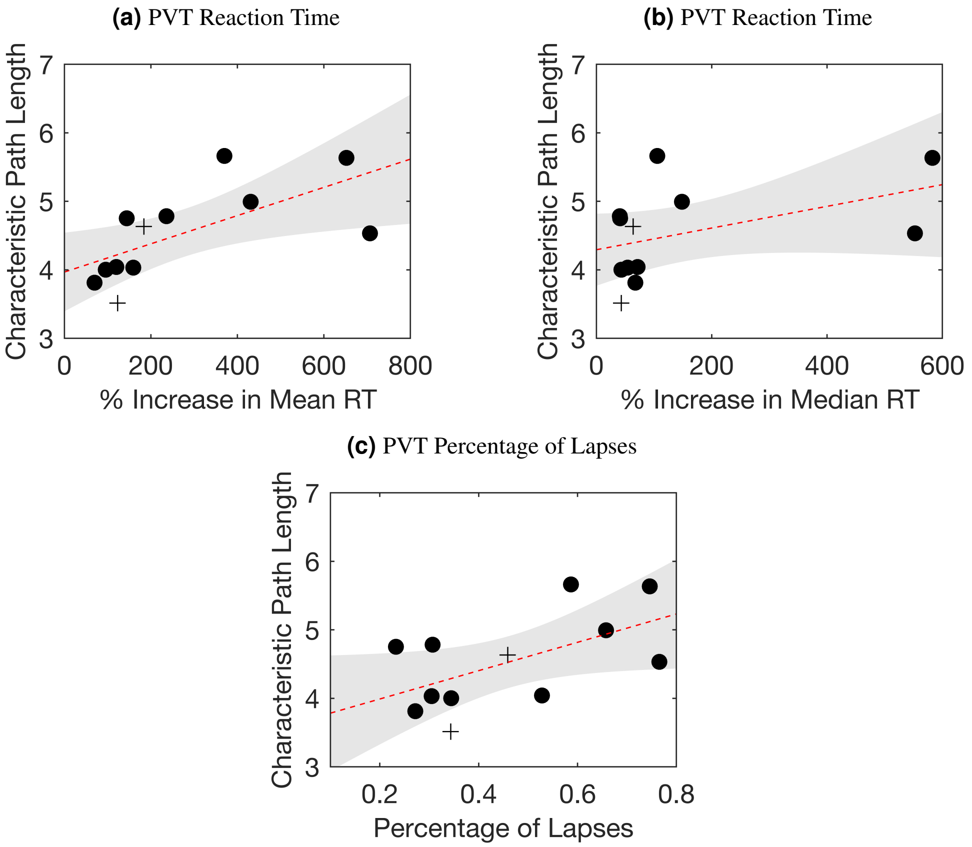

Figure S9. Scatterplot Relating PVT Impairment to B_{-11} Characteristic Path Length. Scatterplots relating the PVT impairment as measured using **(a)** mean RT, **(b)** median RT and **(c)** percentage of lapses to the characteristic path length for the acquisition 11 hours before DLMO. For all plots, participants whose EEG acquisition 11 hours before DLMO is not their first recording are represented by +, while those whose first EEG acquisition is 11 hours before DLMO are denoted by • a black dot, the red dashed line is the linear fit fitted using linear regression between the two variables and the grey patch represents the 95% confidence interval.

Stability

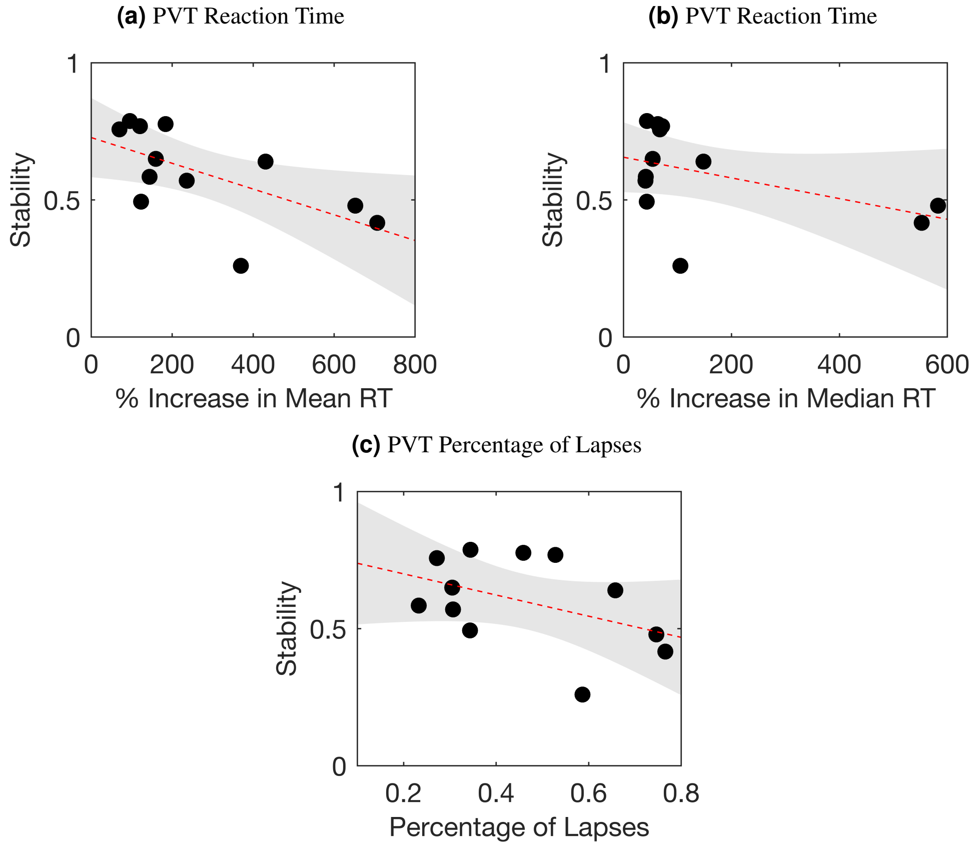

Figure S10. Scatterplot Relating PVT Impairment to B_{FA} Stability. Scatterplots relating the PVT impairment as measured using **(a)** mean RT, **(b)** median RT and **(c)** percentage of lapses to stability for the first acquisition after awakening. For all plots, a black dot represents a participant, the red dashed line is the linear fit fitted using linear regression between the two variables and the grey patch represents the 95% confidence interval.

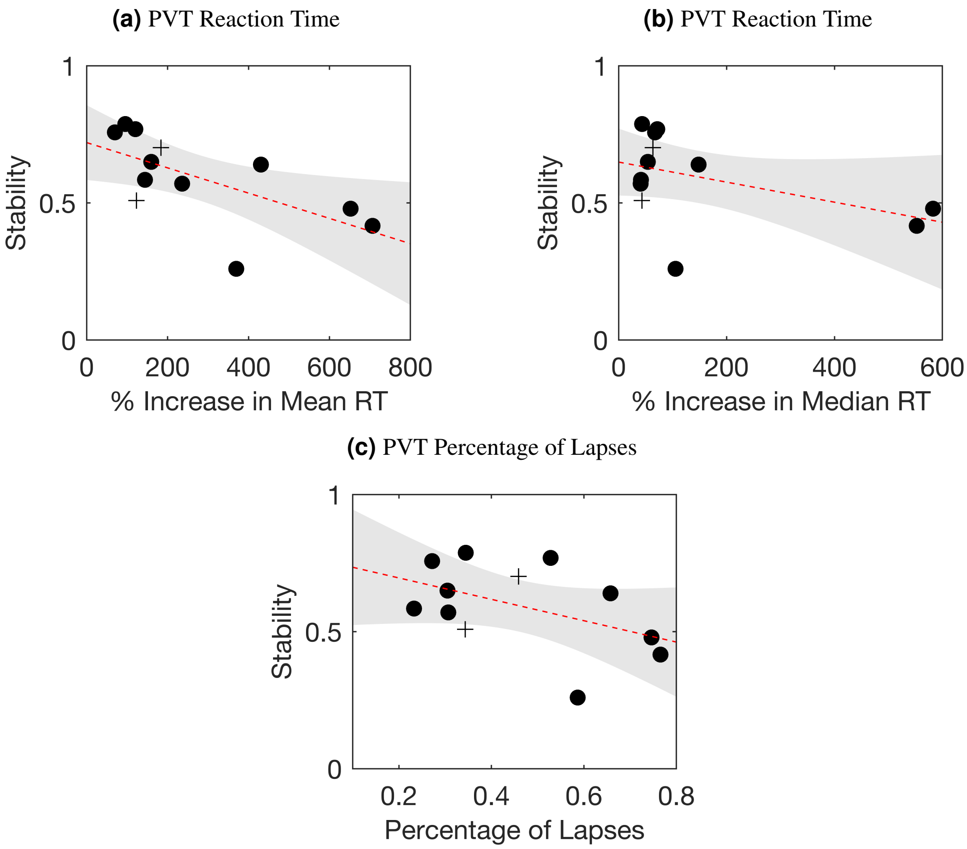

Figure S11. Scatterplot Relating PVT Impairment to B_{-11} Stability. Scatterplots relating the PVT impairment as measured using **(a)** mean RT, **(b)** median RT and **(c)** percentage of lapses to stability for the acquisition 11 hours before DLMO. For all plots, participants whose EEG acquisition 11 hours before DLMO is not their first recording are represented by +, while those whose first EEG acquisition is 11 hours before DLMO are denoted by • a black dot, the red dashed line is the linear fit fitted using linear regression between the two variables and the grey patch represents the 95% confidence interval.

S2 PVT Impairment: No Females

Baseline Acquisition: Graph Metric: PVT Impairment	Correlation	p-value	Adjusted p-value
B_{FA}: $\langle s \rangle$: Mean RT	-0.5289	0.0944	0.2323
B_{FA}: $\langle s \rangle$: Median RT	-0.3239	0.3312	0.3466
B_{FA}: $\langle s \rangle$: $\mathrm{std}_{\mathrm{RT}}$	-0.8555	0.0008	0.0084*
B_{FA}: $\langle s \rangle$: % Lapses	-0.3410	0.3048	0.3466
B_{FA}: $\langle C \rangle$: Mean RT	-0.4248	0.1928	0.3124
B_{FA}: $\langle C \rangle$: Median RT	-0.3585	0.2790	0.3466
B_{FA}: $\langle C \rangle$: $\mathrm{std}_{\mathrm{RT}}$	-0.6109	0.0458	0.1636
B_{FA}: $\langle C \rangle$: % Lapses	-0.3423	0.3028	0.3466
B_{FA} :λ_L: Mean RT	0.5997	0.0511	0.1636
B_{FA} :λ_L: Median RT	0.3961	0.2279	0.3124
B_{FA} :λ_L: $\mathrm{std}_{\mathrm{RT}}$	0.9074	0.0001	0.0037*
B_{FA} :λ_L: % Lapses	0.4381	0.1778	0.3124
B_{FA}: r_s: Mean RT	-0.5840	0.0592	0.1723
B_{FA}: r_s: Median RT	-0.4218	0.1963	0.3124
B_{FA}: r_s: $\mathrm{std}_{\mathrm{RT}}$	-0.7200	0.0125	0.0665
$B_{\text{-}11}$: r_s: % Lapses	-0.4077	0.2132	0.3124
$B_{\text{-}11}$: $\langle s \rangle$: Mean RT	-0.5138	0.1060	0.2422
$B_{\text{-}11}$: $\langle s \rangle$: Median RT	-0.3210	0.3357	0.3466
$B_{\text{-}11}$: $\langle s \rangle$: $\mathrm{std}_{\mathrm{RT}}$	-0.7596	0.0067	0.0535
$B_{\text{-}11}$: $\langle s \rangle$: % Lapses	-0.4014	0.2212	0.3124
$B_{\text{-}11}$: $\langle C \rangle$: Mean RT	-0.3221	0.3340	0.3466
$B_{\text{-}11}$: $\langle C \rangle$: Median RT	-0.2657	0.4297	0.4297
$B_{\text{-}11}$: $\langle C \rangle$: $\mathrm{std}_{\mathrm{RT}}$	-0.3911	0.2343	0.3124
$B_{\text{-}11}$: $\langle C \rangle$: % Lapses	-0.3365	0.3116	0.3466
$B_{\text{-}11}$:λ_L: Mean RT	0.6336	0.0364	0.1636
$B_{\text{-}11}$:λ_L: Median RT	0.4336	0.1828	0.3124
$B_{\text{-}11}$:λ_L: $\mathrm{std}_{\mathrm{RT}}$	0.8664	0.0006	0.0084*
$B_{\text{-}11}$:λ_L: % Lapses	0.5534	0.0774	0.2063
$B_{\text{-}11}$: r_s: Mean RT	-0.5997	0.0511	0.1636
$B_{\text{-}11}$: r_s: Median RT	-0.4272	0.1901	0.3124
$B_{\text{-}11}$: r_s: $\mathrm{std}_{\mathrm{RT}}$	-0.7211	0.0123	0.0665
$B_{\text{-}11}$: r_s: % Lapses	-0.4330	0.1834	0.3124

Table S4. Results of correlating the Baseline Graph Metric with PVT Impairment When Females Were Removed. The Pearson's correlation between the participants' PVT impairment and the graph metric for both baseline acquisitions when correlating mean node strength ($\langle s \rangle$), clustering coefficient ($\langle C \rangle$), characteristic path length (λ_L) and stability (r_s) with the four PVT measures after the two females were removed. The p-values were corrected for multiple comparisons using the Benjamini-Hochberg correction for FDR[2]. * denotes significance p-value < 0.05.

PVT Impairment: No Outliers

For the percentage increase in median RT two participants had an impairment measure more than 1.5 times the interquartile range away from the 75th percentile and were therefore considered outliers. The anlaysis was repeated with these participants removed and the Pearson's correlation and associated p-values are given in Supplementary Table S5.

Baseline Acquisition: Graph Metric: PVT Impairment	Correlation	p-value	Adjusted p-value
B_{FA}: $\langle s \rangle$: Mean RT	-0.6784	0.0310	0.1104
B_{FA}: $\langle s \rangle$: Median RT	-0.3051	0.3914	0.5446
B_{FA}: $\langle s \rangle$: std_{RT}	-0.8524	0.0017	0.0185*
B_{FA}: $\langle s \rangle$: % Lapses	-0.2500	0.4860	0.5888
B_{FA}: $\langle C \rangle$: Mean RT	-0.4195	0.2276	0.4283
B_{FA}: $\langle C \rangle$: Median RT	-0.1983	0.5829	0.6017
B_{FA}: $\langle C \rangle$: std_{RT}	-0.5252	0.1190	0.2611
B_{FA}: $\langle C \rangle$: % Lapses	-0.2177	0.5458	0.5888
B_{FA} :λ_L: Mean RT	0.7552	0.0116	0.0697
B_{FA} :λ_L: Median RT	0.4244	0.2216	0.4283
B_{FA} :λ_L: std_{RT}	0.9006	0.0004	0.0121*
B_{FA} :λ_L: % Lapses	0.3477	0.3249	0.4950
B_{FA}: r_s: Mean RT	-0.5498	0.0996	0.2584
B_{FA}: r_s: Median RT	-0.2193	0.5426	0.5888
B_{FA}: r_s: std_{RT}	-0.7211	0.0186	0.0744
B_{FA}: r_s: % Lapses	-0.2144	0.5520	0.5888
B_{-11}: $\langle s \rangle$: Mean RT	-0.6681	0.0347	0.1112
B_{-11}: $\langle s \rangle$: Median RT	-0.3601	0.3067	0.4950
B_{-11}: $\langle s \rangle$: std_{RT}	-0.7469	0.0131	0.0697
B_{-11}: $\langle s \rangle$: % Lapses	-0.3515	0.3193	0.4950
B_{-11}: $\langle C \rangle$: Mean RT	-0.3570	0.3112	0.4950
B_{-11}: $\langle C \rangle$: Median RT	-0.1787	0.6214	0.6214
B_{-11}: $\langle C \rangle$: std_{RT}	-0.2910	0.4146	0.5528
B_{-11}: $\langle C \rangle$: % Lapses	-0.3098	0.3837	0.55446
B_{-11} :λ_L: Mean RT	0.8194	0.0037	0.0298*
B_{-11} :λ_L: Median RT	0.5428	0.1050	0.2584
B_{-11} :λ_L: std_{RT}	0.8569	0.0015	0.0185*
B_{-11} :λ_L: % Lapses	0.5212	0.1224	0.2611
B_{-11}: r_s: Mean RT	-0.5768	0.0809	0.2352
B_{-11}: r_s: Median RT	-0.2305	0.5218	0.5888
B_{-11}: r_s: std_{RT}	-0.7232	0.0181	0.0744
B_{-11}: r_s: % Lapses	-0.2487	0.4884	0.5888

Table S5. Results of correlating the Baseline Graph Metric with PVT Impairment When Outliers Were Removed. The Pearson's correlation between the participants' PVT impairment and the graph metric for both baseline acquisitions when correlating mean node strength ($\langle s \rangle$), clustering coefficient ($\langle C \rangle$), characteristic path length (λ_L) and stability (r_s) with the four PVT measures after the two outliers were removed. The p-values were corrected for multiple comparisons using the Benjamini-Hochberg correction for FDR[2]. * denotes significance p-value < 0.05.

PVT Performance

Mean Node Strength

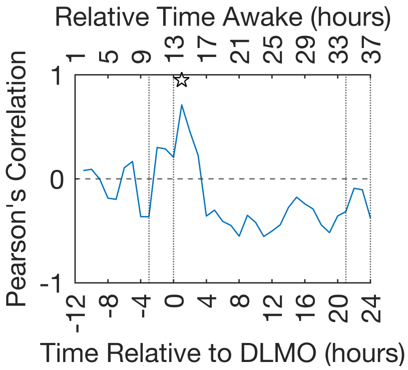

(a) Mean PVT RT (ms)

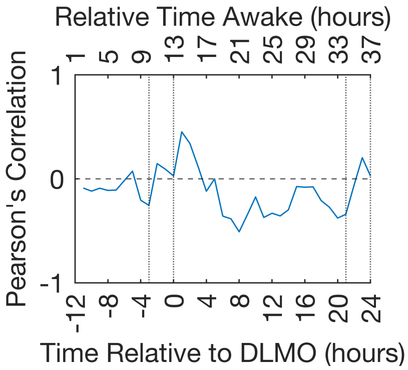

(b) Median PVT RT (ms)

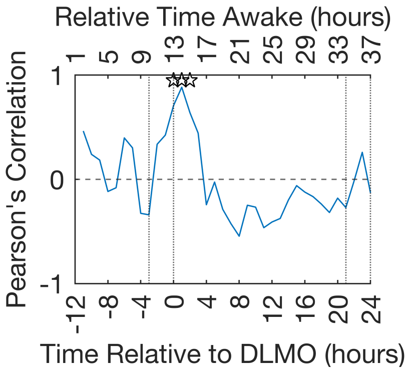

(c) PVT Percentage of Lapses

Figure S12. Pearson's Correlation Between B_{FA} Mean Node Strength and PVT Performance. How the Pearson's correlation coefficient between the baseline mean node strength for the first acquisition after awakening and the PVT performance as measured using **(a)** mean RT, **(b)** median RT and **(c)** **(d)** percentage of lapses changes over the CR. In all plots the ☆ indicates where the *p*-value associated with the correlation is significant $p < 0.05$ (uncorrected) and the dotted vertical lines indicate the times considered to be in the WMZ (3 hours before DLMO to 5 minutes after) and the relative time awake provides an indication of the time awake for the majority of the participants.

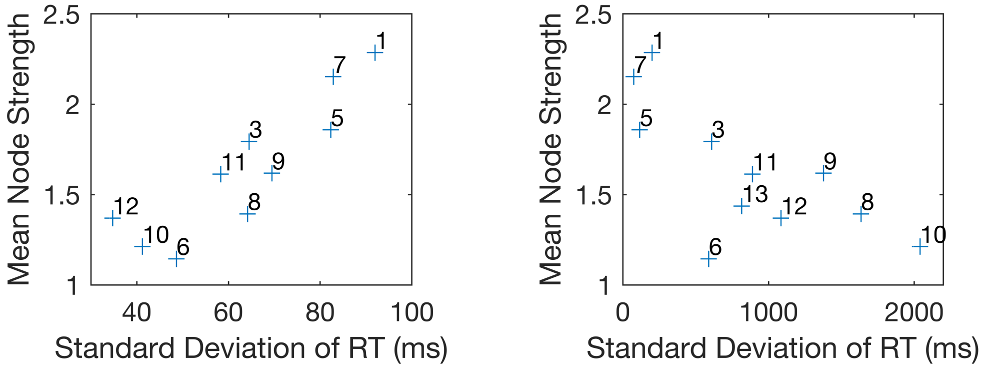

(a) DLMO + 1 h **(b)** DLMO + 11 h

Figure S13. Scatterplots Relating B_{FA} Mean Node Strength to Standard Deviation of RT. The relationship between mean node strength and standard deviation of RT at **(a)** DLMO + 1 h and **(b)** DLMO + 11 h where participants are individually labelled. Note Participant 4 had data missing for these acquisitions.

(a) Mean PVT RT (ms) **(b)** Median PVT RT (ms)

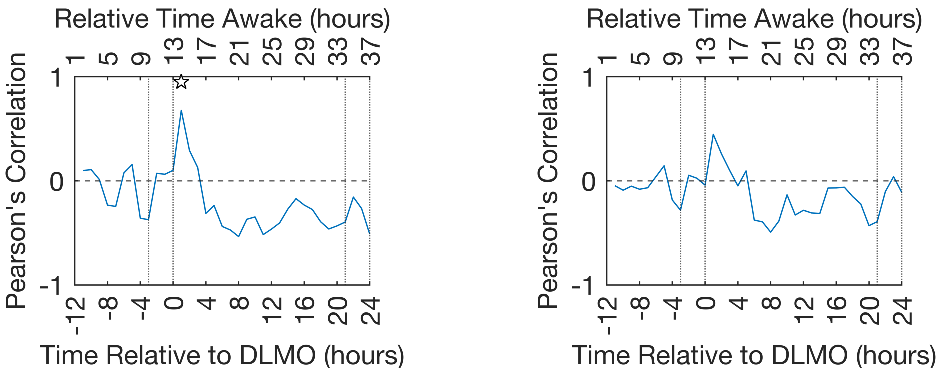

(c) PVT Standard Deviation **(d)** PVT Percentage of Lapses

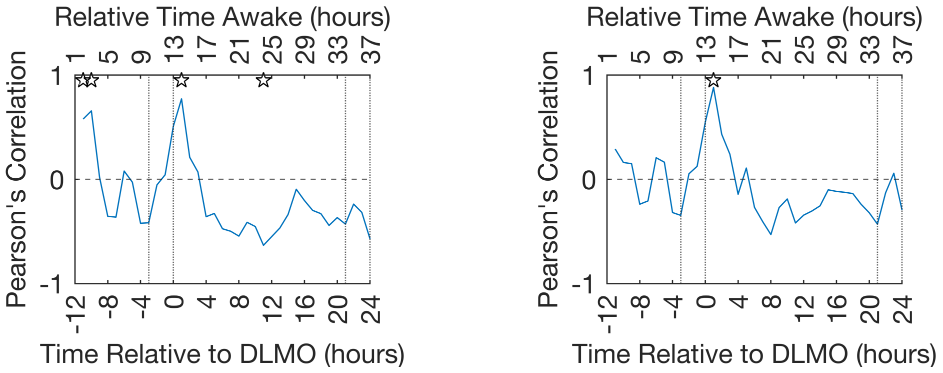

Figure S14. Pearson's Correlation Between B_{-11} Mean Node Strength and PVT Performance. How the Pearson's correlation coefficient between the baseline mean node strength for the acquisition 11 hours before DLMO and the PVT performance as measured using **(a)** mean RT, **(b)**, median RT **(c)** standard deviation of RT and **(d)** percentage of lapses changes over the CR. In all plots the ☆ indicates where the p-value associated with the correlation is significant $p < 0.05$ (uncorrected) and the dotted vertical lines indicate the times considered to be in the WMZ (3 hours before DLMO to 5 minutes after) and the relative time awake provides an indication of the time awake for the majority of the participants.

Clustering Coefficient

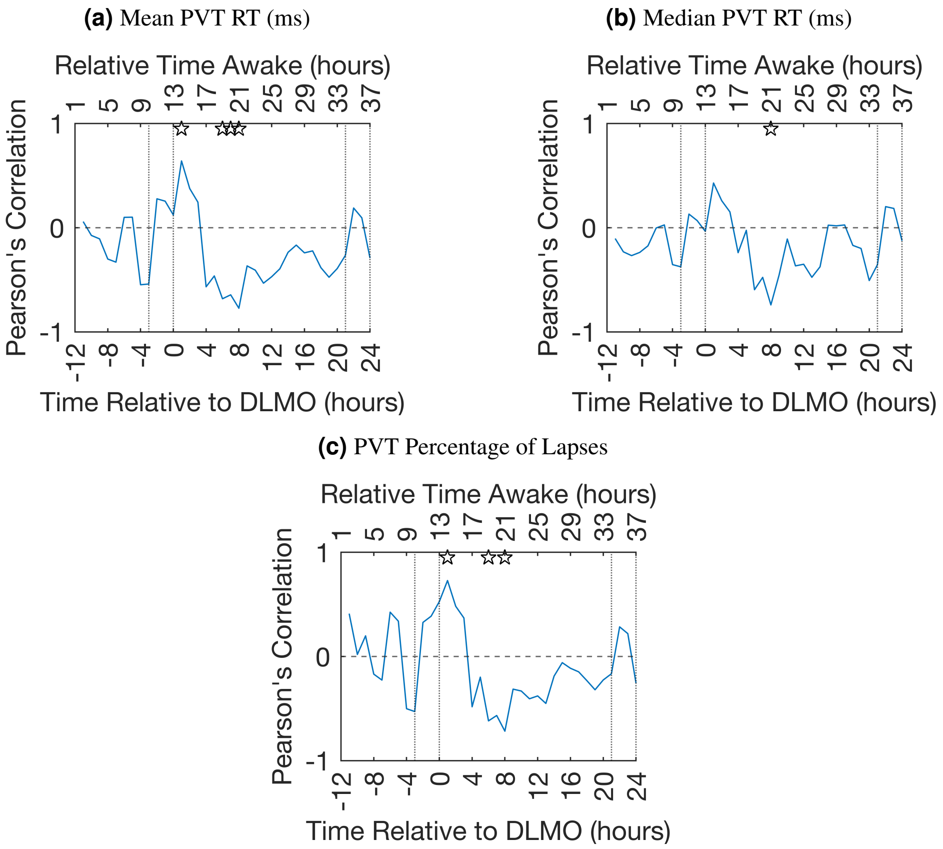

Figure S15. Pearson's Correlation Between B_{FA} Clustering Coefficient and PVT Performance. How the Pearson's correlation coefficient between the baseline clustering coefficient for the first acquisition after awakening and the PVT performance as measured using **(a)** mean RT, **(b)** median RT and **(c)** percentage of lapses changes over the CR. In all plots the ☆ indicates where the p-value associated with the correlation is significant $p < 0.05$ (uncorrected) and the dotted vertical lines indicate the times considered to be in the WMZ (3 hours before DLMO to 5 minutes after) and the relative time awake provides an indication of the time awake for the majority of the participants.

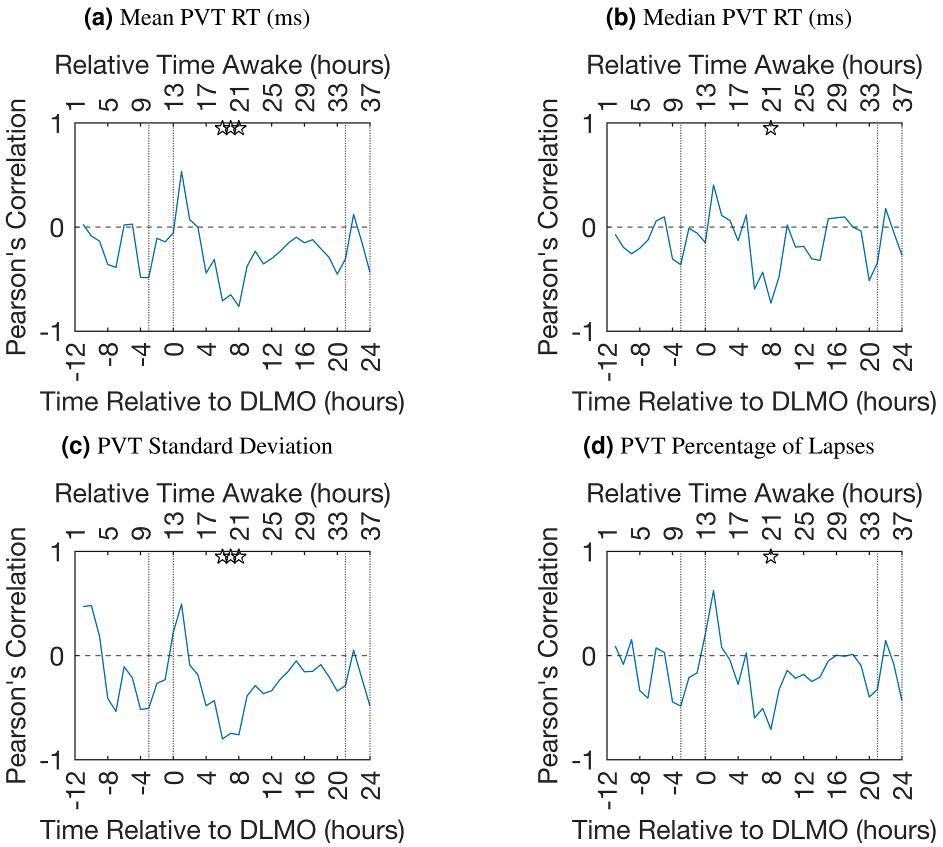

Figure S16. Pearson's Correlation Between B_{-11} Clustering Coefficient and PVT Performance. How the Pearson's correlation coefficient between the baseline clustering coefficient for the acquisition 11 h before DLMO and the PVT performance as measured using **(a)** mean RT, **(b)** median RT, **(c)** standard deviation of RT and **(d)** percentage of lapses changes over the CR. In all plots the ☆ indicates where the *p*-value associated with the correlation is significant $p < 0.05$ (uncorrected) and the dotted vertical lines indicate the times considered to be in the WMZ (3 hours before DLMO to 5 minutes after) and the relative time awake provides an indication of the time awake for the majority of the participants.

Characteristic Path Length

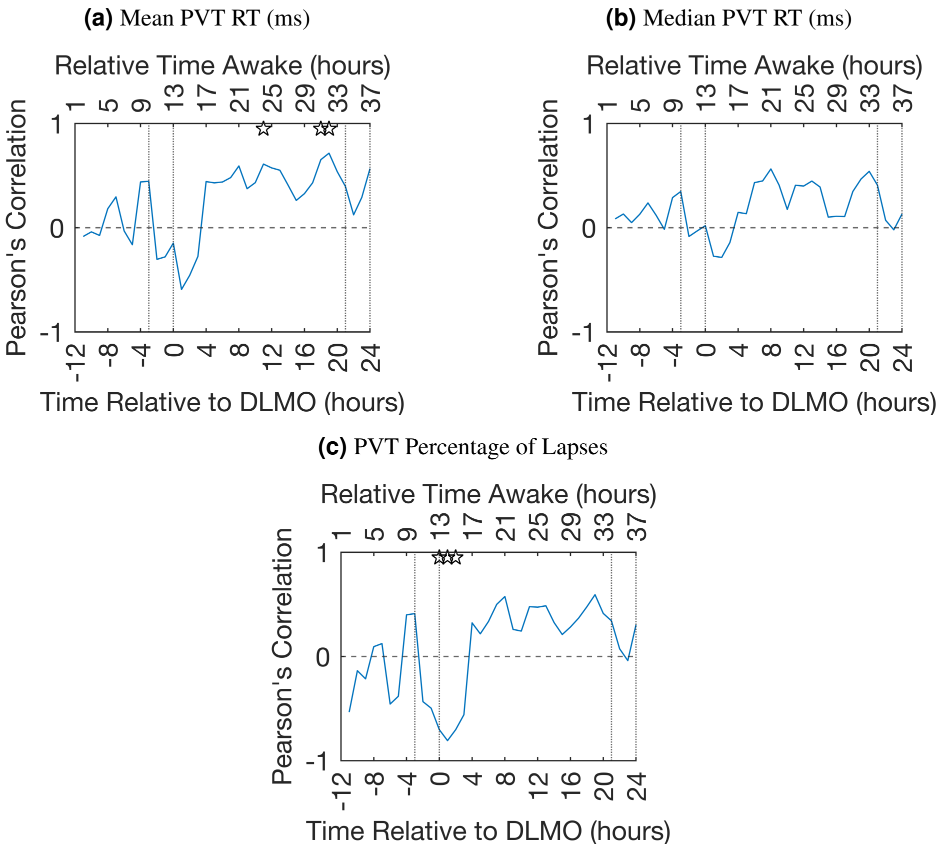

Figure S17. **Pearson's Correlation Between B_{FA} Characteristic Path Length and PVT Performance.** How the Pearson's correlation coefficient between the baseline characteristic path length for the first acquisition after awakening and the PVT performance as measured using **(a)** mean RT, **(b)** median RT and **(c)** percentage of lapses changes over the CR. In all plots the ☆ indicates where the *p*-value associated with the correlation is significant $p < 0.05$ (uncorrected) and the dotted vertical lines indicate the times considered to be in the WMZ (3 hours before DLMO to 5 minutes after) and the relative time awake provides an indication of the time awake for the majority of the participants.

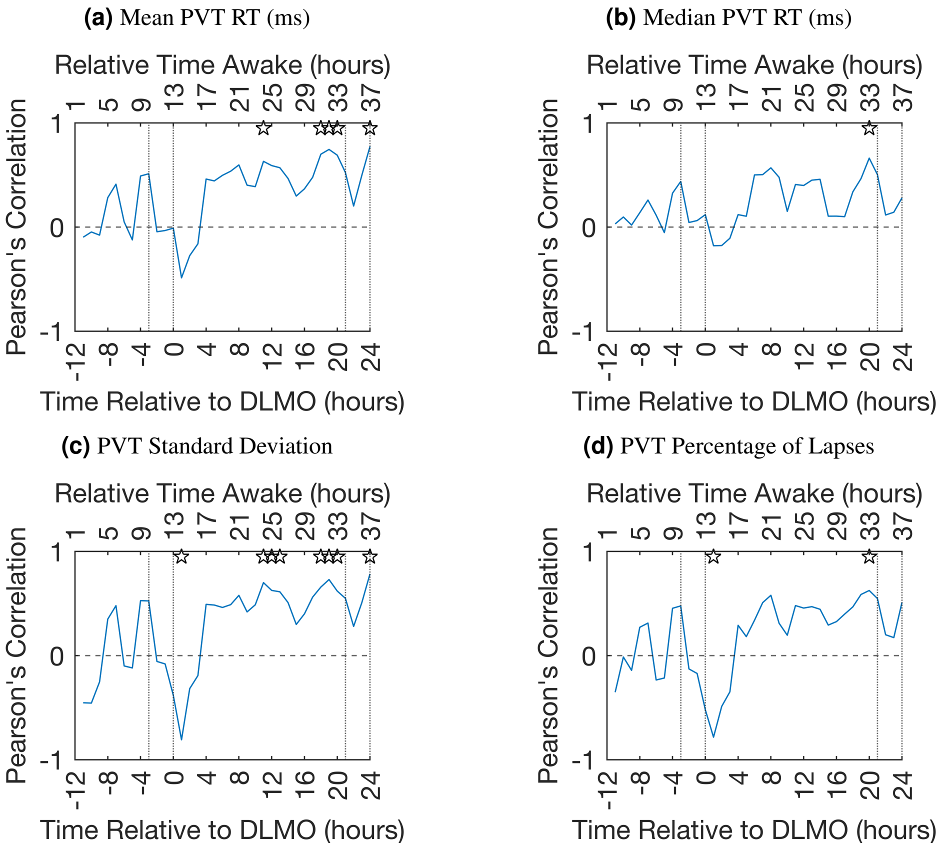

Figure S18. **Pearson's Correlation Between B_{-11} Characteristic Path Length and PVT Performance.** How the Pearson's characteristic path length between the baseline clustering coefficient for the acquisition 11 h before DLMO and the PVT performance as measured using **(a)** mean RT, **(b)** median RT, **(c)** standard deviation of RT and **(d)** percentage of lapses changes over the CR. In all plots the ☆ indicates where the *p*-value associated with the correlation is significant $p < 0.05$ (uncorrected) and the dotted vertical lines indicate the times considered to be in the WMZ (3 hours before DLMO to 5 minutes after) and the relative time awake provides an indication of the time awake for the majority of the participants.

Stability

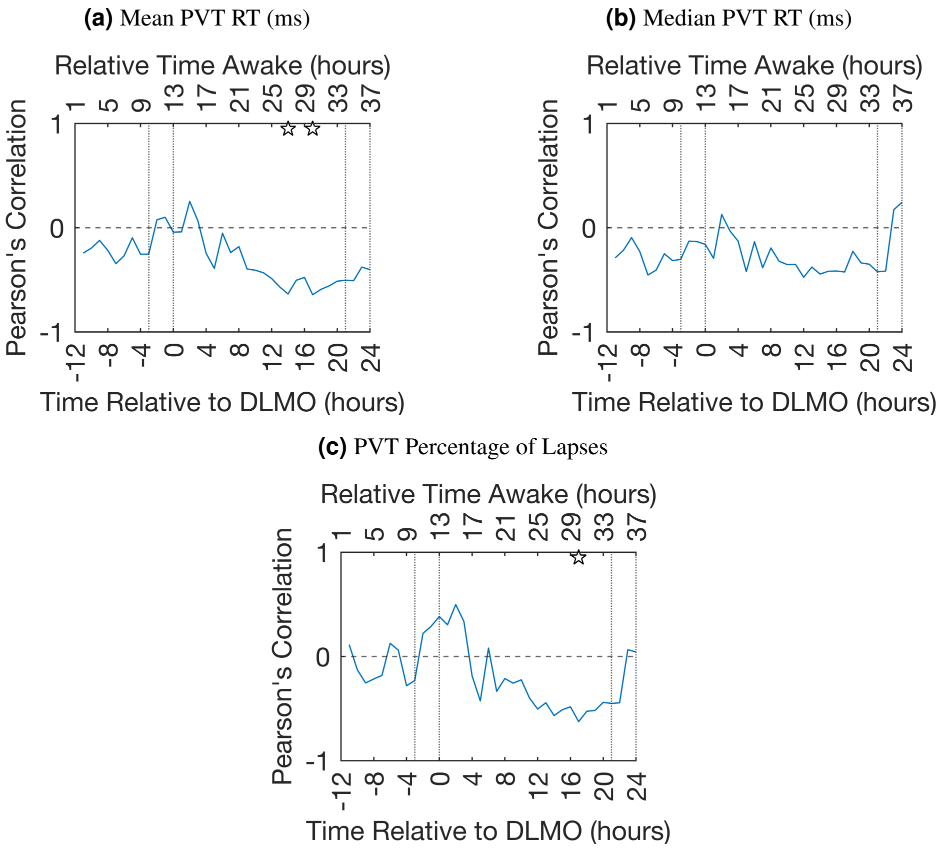

Figure S19. **Pearson's Correlation Between B_{FA} Stability and PVT Performance.** How the Pearson's correlation coefficient between the baseline stability for the first acquisition after awakening and the PVT performance as measured using **(a)** mean RT, **(b)** median RT and **(c)** percentage of lapses changes over the CR. In all plots the ☆ indicates where the *p*-value associated with the correlation is significant $p < 0.05$ (uncorrected) and the dotted vertical lines indicate the times considered to be in the WMZ (3 hours before DLMO to 5 minutes after) and the relative time awake provides an indication of the time awake for the majority of the participants.

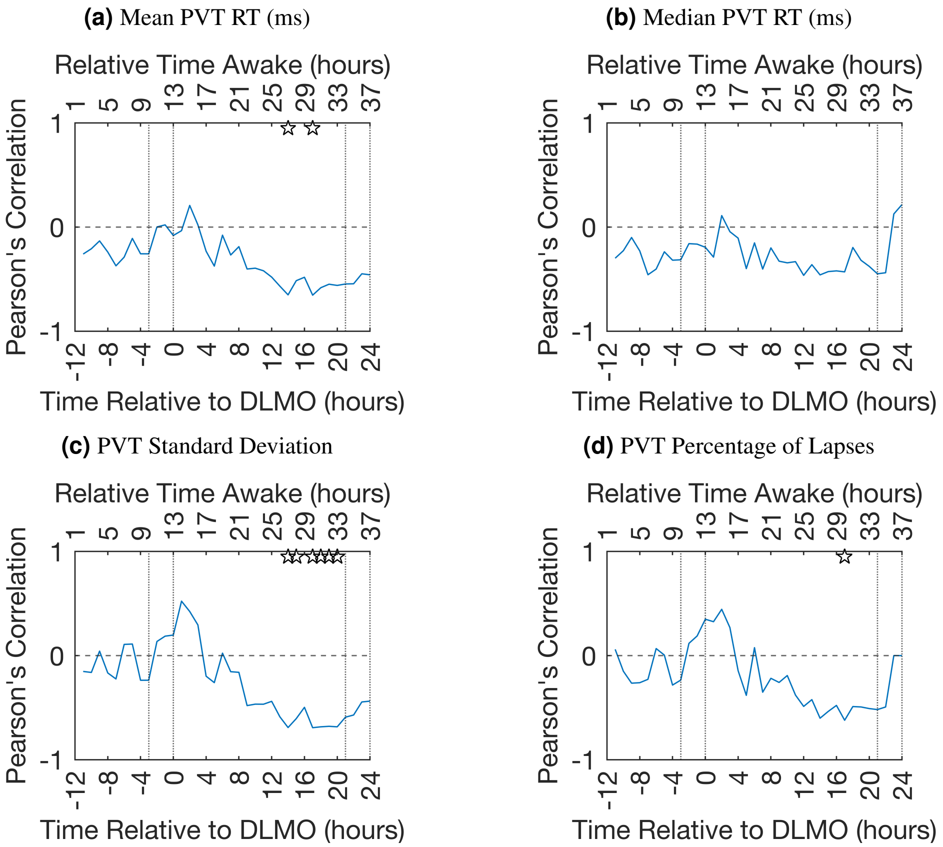

Figure S20. Pearson's Correlation Between B_{-11} Stability and PVT Performance. How the Pearson's correlation coefficient between the baseline stability for the acquisition 11 h before DLMO and the PVT performance as measured using **(a)** mean RT, **(b)** median RT, **(c)** standard deviation of RT and **(d)** percentage of lapses changes over the CR. In all plots the ☆ indicates where the *p*-value associated with the correlation is significant $p < 0.05$ (uncorrected) and the dotted vertical lines indicate the times considered to be in the WMZ (3 hours before DLMO to 5 minutes after) and the relative time awake provides an indication of the time awake for the majority of the participants.

Stability

Group Level

Frequency Band	Paired Cohen's d	p-value	Adjusted p-value
Delta	0.0056	0.8238	0.8238
Theta	0.2802	0.1384	0.1846
Alpha	0.4252	0.0680	0.1846
Beta	0.3965	0.1333	0.1846

Table S6. Stability Prior to and During the WMZ. Cohen's d effect size and associated p-values for paired t-tests for each frequency band. The tests compared the mean stability for 3 hours prior to the WMZ to the WMZ with the mean stability for the participant over the 3 hours during the WMZ. Both p-values and adjusted p-values corrected for multiple comparisons using the Benjamini-Hochberg method are given[2].

PVT Groups

Frequency Band	DLMO-11h - DLMO	DLMO+1h - DLMO+12h	DLMO+13h - DLMO+24h
Delta	0.0475* (0.0317)	0.0839 (0.0629)	0.3882 (0.3882)
Theta	< 0.0001* (< 0.0001)	0.3519 (0.3226)	0.0108* (0.0054)
Alpha	< 0.0001* (< 0.0001)	0.0161* (0.0094)	0.0006* (0.0002)
Beta	< 0.0001* (< 0.0001)	0.0012* (0.0005)	0.1794 (0.1495)

Table S7. Table Showing the *p*-values for Permutation Tests Comparing Stability with Participants Grouped by Standard Deviation of RT. Results for permutation tests ($n = 10,000$) comparing the mean value of the low and high impairment groups over adjacent 12 hour periods. The p-values have been corrected using the Benjamini-Hochberg method of correcting for FDR[2]. * denotes significance p-value < 0.05. Uncorrected p-values are given in brackets.

Individual

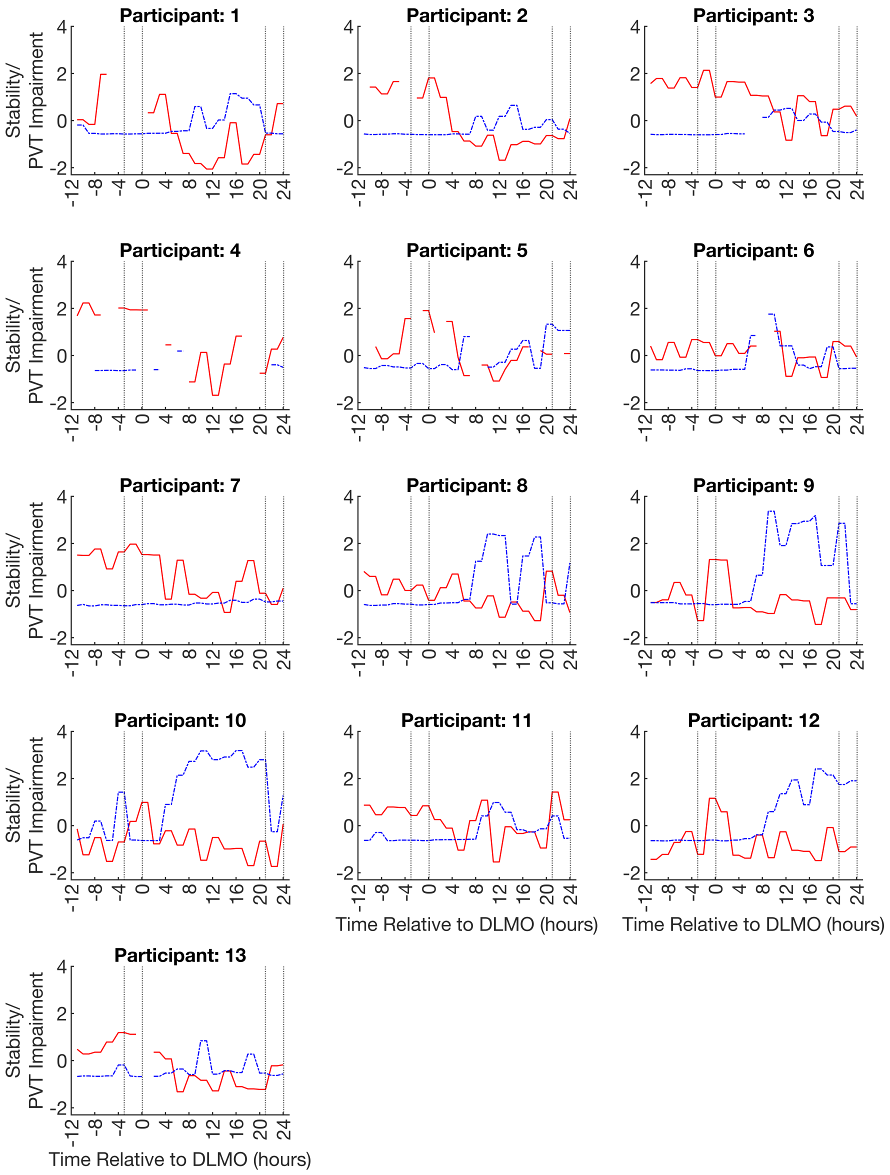

Figure S21. Individual Stability and Standard Deviation on PVT. The individual time-series for the standard deviation of RT on the PVT (-·-·-·-) and the stability of the FNs (——). The dotted vertical lines indicate the times considered to be in the WMZ (3 hours before DLMO to 5 minutes after).

Participant	Impairment Group	Spearman's Rank Correlation	p-value	Adjusted p-value
1	Low	-0.6642	< 0.0001	0.0003*
2	Low	-0.8117	< 0.0001	< 0.0001*
3	Low	-0.7577	< 0.0001	< 0.0001*
4	Low	-0.8137	0.0042	0.0078*
5	Low	-0.4035	0.0292	0.0421*
6	High	-0.1545	0.3823	0.4147
7	Low	-0.5801	0.0002	0.0007*
8	High	-0.5398	0.0007	0.0018*
9	High	-0.3262	0.0522	0.0678
10	High	-0.2083	0.2228	0.2633
11	NA	-0.3684	0.0270	0.0421*
12	High	0.0010	0.9952	0.9952
13	High	-0.5287	0.0013	0.0028*

Table S8. Spearman's Rank Correlation Between std RT and Stability for Individuals. The Spearman's rank correlation and associated p-value for the correlation between the participants standard deviation of RT and the stability of their FNs over the CR. The p-values have been corrected using the Benjamini-Hochberg method of correcting for FDR[2]. * denotes significance p-value < 0.05.

Stability: Additional Participants

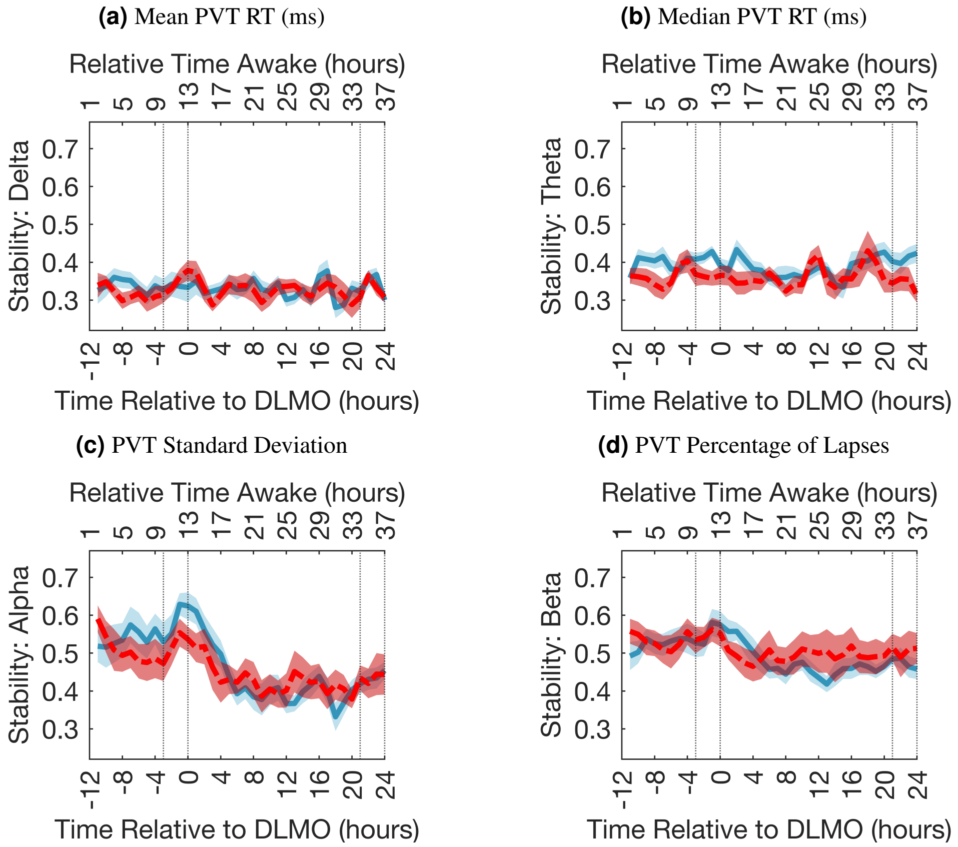

Figure S22. **Stability of Participants FN's: Additional Participants.** The median stability, as measured from correlating the PLF networks from all epochs for that participant from an EEG acquisition for a given frequency band **(a)** Delta, **(b)** Theta, **(c)** Alpha and **(d)** Beta. The solid blue line (——) is the mean across the median stability of the 13 participants in this study and the dashed red line (- - - - -) is the mean across the median stability of the 9 additional participants. The corresponding blue and red shaded area is the standard error of the mean. The dotted vertical lines indicate the times considered to be in the WMZ (3 hours before DLMO to 5 minutes after).

References

1. McMahon, W. R. *et al.* The impact of structured sleep schedules prior to an in-laboratory study: Individual differences in sleep and circadian timing. *PLOS ONE* **15**, DOI: 10.1371/journal.pone.0236566 (2020).

2. Groppe, D. M. *fdr_bh* (MATLAB Central File Exchange, 2023). https://www.mathworks.com/matlabcentral/fileexchange/27418-fdr_bh.
